# Bacterial diversity differences along an epigenic cave stream reveal evidence of community dynamics, succession, and stability

**DOI:** 10.3389/fmicb.2015.00729

**Published:** 2015-07-21

**Authors:** Kathleen Brannen-Donnelly, Annette S. Engel

**Affiliations:** Department of Earth and Planetary Sciences, University of TennesseeKnoxville, TN, USA

**Keywords:** community assembly, stream, cave, microorganisms, succession, community dynamics, Bio-Traps®

## Abstract

Unchanging physicochemical conditions and nutrient sources over long periods of time in cave and karst subsurface habitats, particularly aquifers, can support stable ecosystems, termed autochthonous microbial endokarst communities (AMEC). AMEC existence is unknown for other karst settings, such as epigenic cave streams. Conceptually, AMEC should not form in streams due to faster turnover rates and seasonal disturbances that have the capacity to transport large quantities of water and sediment and to change allochthonous nutrient and organic matter sources. Our goal was to investigate whether AMEC could form and persist in hydrologically active, epigenic cave streams. We analyzed bacterial diversity from cave water, sediments, and artificial substrates (Bio-Traps®) placed in the cave at upstream and downstream locations. Distinct communities existed for the water, sediments, and Bio-Trap® samplers. Throughout the study period, a subset of community members persisted in the water, regardless of hydrological disturbances. Stable habitat conditions based on flow regimes resulted in more than one contemporaneous, stable community throughout the epigenic cave stream. However, evidence for AMEC was insufficient for the cave water or sediments. Community succession, specifically as predictable exogenous heterotrophic microbial community succession, was evident from decreases in community richness from the Bio-Traps®, a peak in Bio-Trap® community biomass, and from changes in the composition of Bio-Trap® communities. The planktonic community was compositionally similar to Bio-Trap® initial colonizers, but the downstream Bio-Trap® community became more similar to the sediment community at the same location. These results can help in understanding the diversity of planktonic and attached microbial communities from karst, as well as microbial community dynamics, stability, and succession during disturbance or contamination responses over time.

## Introduction

Caves are diagnostic dissolutional features in karst landscapes underlain by soluble rock (e.g., limestone or dolomite) where surface water sinks into the subsurface and flows in a network of self-evolving underground stream passages (Ford and Williams, 2013). Although hydrological flow regimes, watershed geometry, aqueous geochemistry, and bedrock geology differ between karst systems (Nico et al., 2006; Simon et al., 2007; Bonacci et al., 2008), many have similar, stable environmental conditions and components that contribute to habitability and ecosystem development (Hahn and Fuchs, 2009; Griebler et al., 2010). Microbes are important components of all subterranean ecosystems (Chapelle, 2000) and of every type of karst habitat (Griebler and Lueders, 2009). Although the compositions of microbial communities (from the aspect of alpha-diversity) have been widely evaluated from karst (Griebler and Lueders, 2009), much still remains to be explored, including microbial diversity trends over time (Engel, 2010). Microbes regulate chemical reactions that cause mineral dissolution and precipitation (Engel et al., 2004; Engel and Randall, 2011; Lian et al., 2011) and affect contaminant remediation (Thomas and Ward, 1992). As such, interest in microbial communities in various karst settings has increased (Griebler et al., 2010), and attempts have been made to understand whether microbial diversity differs throughout distinct types of karst systems and what ecosystem conditions control or regulate community composition. For instance, in karst aquifers and cave pools where water residence times are exceedingly long, from months to years, autochthonous microbial endokarst communities (AMEC) develop (Farnleitner et al., 2005; Pronk et al., 2008). Understanding AMEC is important to groundwater ecology, biogeochemistry of karst aquifers, and water resource management and conservation (Farnleitner et al., 2005; Pronk et al., 2008; Griebler and Lueders, 2009; Zhou et al., 2012).

Previously described AMEC have been sampled as planktonic phenomena from annual and monthly sample events of karst springs (Farnleitner et al., 2005; Pronk et al., 2008). A uniform definition for AMEC has not been applied, despite other types of groundwater systems having taxonomic and functionally distinct attached and planktonic communities (Hazen et al., 1991; Alfreider et al., 1997; Lehman, 2007; Flynn et al., 2008; Zhou et al., 2012). Conceptually AMEC should be homogenized communities of planktonic and attached microbial cells from within a karst aquifer setting. Under elevated flow conditions during recharge events, high flow velocities would mobilize sediment (Dogwiler and Wicks, 2004) and cause high shear stress on sediment-attached cells (Rehmann and Soupir, 2009; Ghimire and Deng, 2013). Biofilm development on sediments and aquifer surfaces would be limited and attached cells would become entrained into the water column and become part of the planktonic community (Rehmann and Soupir, 2009). A prescribed minimum time limit for AMEC formation in karst has not been described, but this is not surprising because the stability and potential AMEC successional patterns over time in most groundwater systems are also not well understood (Farnleitner et al., 2005). Typical AMEC bacterial compositions are apparently comprised of Acidobacteria, Nitrospira, Gammaproteobacteria, and Deltaproteobacteria, and AMEC comprise the majority of the overall community abundances (Farnleitner et al., 2005; Pronk et al., 2008). The major taxonomic groups in AMEC are phylogenetically related to surface-derived groups, but not identical, thereby highlighting the importance of being sourced from within a subsurface system. Although no truly endemic karst microorganisms have been identified (Griebler and Lueders, 2009), arguably enhanced genetic divergences between surface communities and AMEC could result from long flow path travel distances and longer periods of isolation between the surface and subsurface.

As such, it is unclear whether AMEC are present or can persist in systems where turnover rates are expected to be high, such as in cave streams. Cave streams are dynamic, usually turbulent underground features that form from sinking surface water. Water is sourced from the surface and may reemerge from a conduit some distance later as a spring. Cave stream habitats that become established based on prevailing physicochemical gradients may only last for hours to weeks, according to the hydrological connection (i.e., continuous, flashy, etc.) with the surface. Sediment suspension and deposition events caused by recharge flooding or flushing of the system could compositionally homogenize water and sediment microbial communities (at the level of beta-diversity), which would hamper the ability to detect AMEC from transported allochthonous communities. In this study, we investigated the diversity and prevalence of microorganisms from 16S rRNA gene sequences in stream water and cave sediments along a continuously flowing cave stream of fixed length but having different flow rates due to storm events over a 6 month period. In addition to documenting novel bacterial diversity for an epigenic cave stream, we compared water-transported (i.e., planktonic) and sediment (i.e., attached) bacterial diversity to test the hypothesis that an AMEC exists, despite storm water and sediment disturbances and differential contribution of surface-derived bacterial groups into the subsurface. We expected water and sediment communities to be similar to each other after high flow events, but that sediment communities would represent AMEC in between high flow events that would resuspend some or all of the cave sediments.

We also hypothesized that disturbance events reveal successional patterns between upstream and downstream communities. Studying microbial community successional patterns has proven difficult in many ecological systems (Shade et al., 2013). For this study, we used the definition of succession from Fierer et al. (2010), as the “orderly and predictable manner by which communities change over time following the colonization of a new environment.” During 4 months, we seeded bacterial communities on artificial substrates (Bio-Trap® samplers) that were fixed in one upstream and one downstream location in the cave system. The Bio-Traps® were subsampled every month so that only a portion of material was removed and the rest remained in a sampler. This experimentally contrasted cave stream sediment samples, which had the potential to be redistributed and mobilized during the study. The newly formed Bio-Trap® communities every month were compared to preexisting water and sediment communities to test the hypothesis that Bio-Trap® communities would resemble sediment communities over time, despite being colonized initially by planktonic microbes. Combined, these results provide evidence for cave stream community assembly and community succession. Underlying drivers that could explain spatial and temporal changes in bacterial diversity were statistically evaluated against stream discharge, rainfall, and geochemistry, including fluorescence spectral data for chromophoric dissolved organic matter (CDOM) that highlighted organic matter seasonal changes.

## Materials and methods

### Site characterization

**Figure 1:**
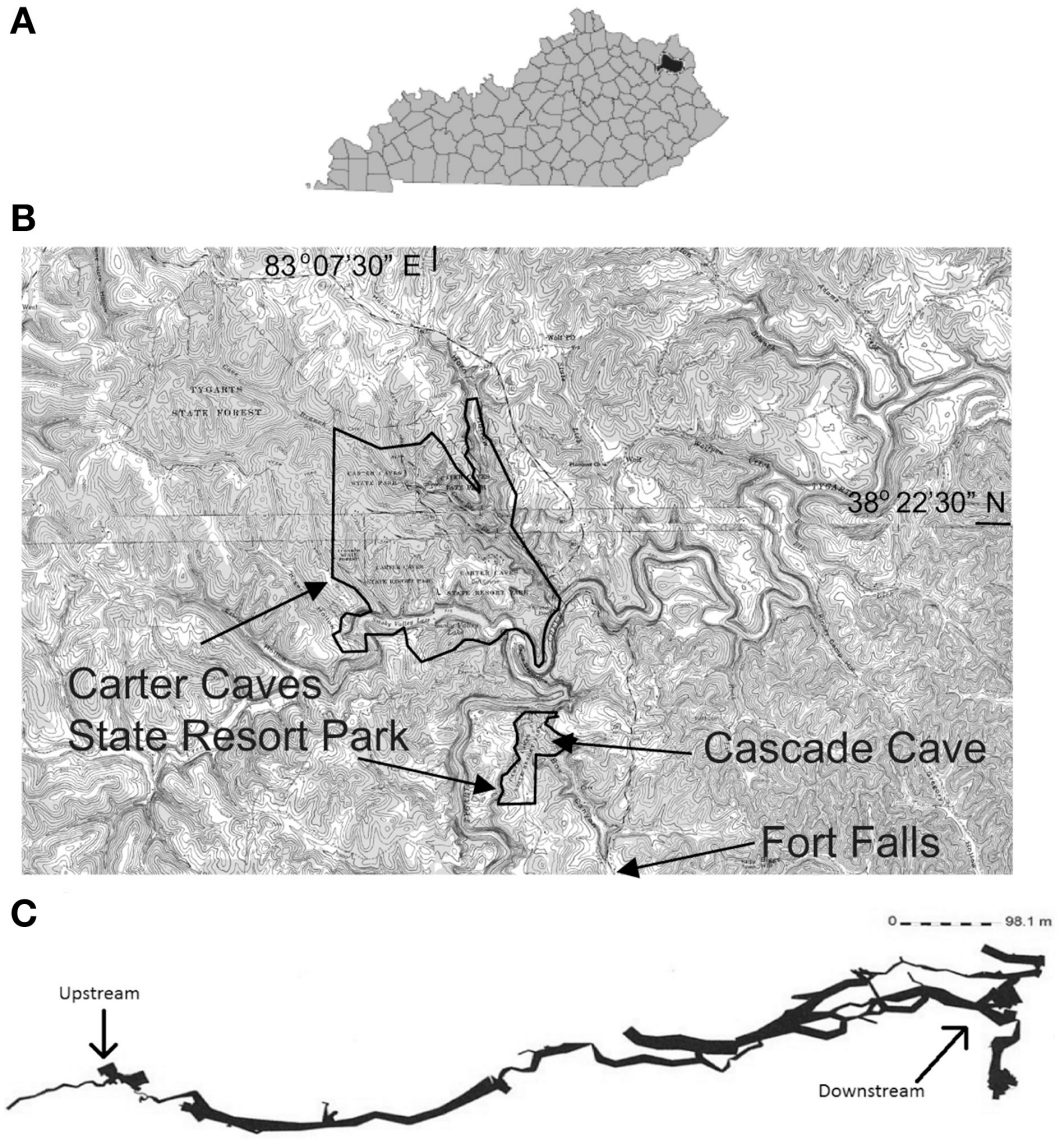
**(A)** Black area denotes Carter County, Kentucky. **(B)** Spliced topographic maps from the United States Geological Survey showing the location of Carter Caves State Resort Park boundaries, relative location of Cascade Cave and Fort Falls, modified from Engel and Engel (2009). Specific location details are withheld at the request of the park. **(C)** A generalized line-plot map of the Cascade Cave system, including Cascade Cave (downstream), Sandy Cave, and Jones Cave (upstream). Map provided by Dr. Horton H. Hobbs, III, and the Wittenberg University Speleological Society, Springfield, Ohio (USA).

We conducted the study from July through December, 2013, in the Cascade Cave system within Carter Caves State Resort Park (CCSRP) in Carter County, Kentucky (Figure 1). The system is comprised of at least three surveyed caves that formed within the carbonate Slade Formation (Mississippian) (Engel and Engel, 2009). The caves are situated in the James Branch stream watershed, which flows into Tygart's Creek at local base-level (Dougherty, 1985; Engel and Engel, 2009). The entire watershed is approximately 4 km^2^. The surface stream flows over Pennsylvanian and Mississippian interbedded sandstone and shale units before it sinks underground at a waterfall called Fort Falls (herein referred to as the surface sampling location). The cave system has flowing water year-round. Jones Cave is the first access point to the cave stream (herein referred to as the upstream sampling location). There is a karst window 500 m downstream from Jones Cave where surface water enters the subsurface from a small surface stream. The entrance to another cave, Sandy Cave, is located at the window. Cascade Cave has several entrances, and one is reached downstream of the karst window and Sandy Cave. Where the cave stream emerges at the surface as a karst spring and another entrance to Cascade Cave, we sampled at the Lake Room (herein referred to as the downstream sampling location). The total estimated distance of the underground cave stream from the top of the water fall to resurgence is approximately 1.5 km. Preliminary (i.e., unpublished) tracer tests from Fort Falls to the Lake Room indicate a base flow travel time of about 12 h. All of the sampling was done in less than 3 h to evaluate contemporaneous microbial communities that could be present or established at each location, specifically planktonic communities from water, attached communities from sediment, and newly formed communities from the Bio-Trap® devices.

At each sample location and time, water flow rates were calculated by an average of three flow readings using a Geopacks Basic Flowmeter. Passage or channel cross-sectional area and water depth were measured to calculate discharge (Q) as the product of velocity, depth of the water, and channel width. Sediment particle transport was calculated by comparing stream velocity to the Stokes Settling Velocity for all the grain sizes present in the sediment samples (methods describes below), according to Ferguson and Church (2004). With no automated meteorological station data from CCSRP, daily precipitation data are measured and recorded at the Fort Falls location by a citizen scientist who works in CCSRP (Supplemental Figure 1).

### Water and sediment sampling and analyses

At each sampling location, physicochemical properties were measured using standard electrode methods (American Public Health et al., 2005), including pH, temperature, dissolved oxygen, total dissolved solids, and conductivity. At least 500 mL of cave stream water were manually filtered through duplicate 0.22 μm Sterivex™ (PVDF, EMD Millipore) filters. Filters were frozen at −20°C until use. The filtered water was collected for anion (using clean HDPE bottles), cation (using acid-washed HDPE bottles), and total organic carbon (TOC) and total nitrogen (TN) analyses (using baked glass VOA vials). Cations were preserved with trace metal grade nitric acid. Samples were put on ice for transport and stored at 4°C until analysis. Alkalinity, representing bicarbonate concentration, was measured from 0.2 μm-filtered water in the field by manual titration to an end-point of pH 4.3 with 0.1 N H_2_SO_4_ (American Public Health et al., 2005). Major dissolved ions were measured on a Dionex ICS-2000 ion chromatograph, with standards checks accurate within two standard deviations. Total inorganic carbon (TIC) and dissolved organic carbon (DOC) concentrations were analyzed for filtered water with a Shimadzu Model TOC-V Total Carbon Analyzer. DOC was reported as the difference between dissolved non-purgable organic carbon and TIC (American Public Health et al., 2005). The standard used for minimum detection limit was C_8_H_5_KO_4_, and the precision between replicate sample injections was 2% of the relative percent difference (RPD) for DOC > 4 mg/L and 5% RPD for DOC < 4 mg/L. TN content were measured by a high temperature catalytic oxidation with chemiluminescence minimum detection level of 0.01 mg/L (ASTM, 2008).

During some sampling times, only bare carbonate rock was exposed at a sample location in the cave where sediment had been present previously. If sediments were available to collect at a sampling location, then at least 25 g were aseptically collected from 0 to 2 cm deep and placed into sterile Falcon tubes; as such, any one particle had to be <20 mm to fit in the tube. Sediment was stored at −20°C until use. Sediment grain size was analyzed in triplicate for each sample from sieving air-dried material through sieves for >2, 1 mm, 500, 250, 150, and <150 μm. Weights of each sieved aliquot were measured to ±0.0001 g at least three times.

### Fluorescence spectroscopy

Qualitative information about organic matter sources, composition, bioavailability, and the differences between allochthonous and autochthonous DOM can be determined from the natural concentration of CDOM (Coble, 1996; McKnight et al., 2001). The relative contributions of different CDOM sources in the filtered stream water were evaluated from excitation emission matrices (EEMs) produced by a Horiba Scientific Fluoromax4 spectrofluorometer with a Xenon lamp. A total of 43 emission scans were completed for each sample with setting of λEM = 250–550-nm, 2.5-nm steps; λEX = 240–550-nm, and 5-nm steps. Instrument settings were PMT voltage 800 V, EX/EM slits 5-nm each, and an integration time 0.1 s. Spectral corrections for primary and secondary inner filter effects of EEMs were made using absorbance spectra collected using a Thermo Scientific Evolution 200 series spectrophotometer in a 1-cm cuvette over the 200–700 nm wavelength range with pyrogen-free deionized (DI) (>18.1 MΩ) water as the reference. Raman scattering was removed from EEMs by subtracting a DI water blank spectrum collected from each sample spectrum. Rayleigh scattering effects were edited from each spectrum, following correction and blank subtraction (Lakowicz, 2007).

Fluorescence data were interpreted from index analyses from individual emission scans or extracted from EEMs using methods previously described (Birdwell and Engel, 2010). We used the Fluorescence Index (FI) to assess terrestrial and microbial contributions to CDOM fluorescence (McKnight et al., 2001), the Humification Index (HIX) to estimate the degree of DOM humification (Ohno, 2002), and the Biological or Freshness Index (BIX) to evaluate the contribution of biological or microbial processes to CDOM fluorescence (Huguet et al., 2009).

### Microbial succession experiment

Standard Bio-Trap® samplers baited with 30 g of 2-mm diameter Bio-Sep® beads made of Nomex® composite and powdered activated carbon were obtained from Microbial Insights, Inc. (Knoxville, TN, USA) (www.microbe.com). Slits on the samplers were 0.4 mm wide, and the inside of the samplers was wrapped with 0.011 mm mesh screen to reduce sediment and macrofauna intrusion. Bio-Traps® were suspended in triplicate (overall weight 1.3 kg) via ropes attached to the cave wall by using non-destructive, spring-loaded camming devices at Jones Cave (upstream location) and in the Lake Room (downstream location) (Supplemental Figure 1). At base-flow (i.e., low flow) conditions, Bio-Traps® were in contact with sediment or bare rock at the bottom of the stream channel, but were not buried in the sediment. The samplers were also weighted by using 0.2 kg weights so that they would become suspended in the water column only during exceptionally high flow events (i.e., in excess of 0.5 m/s). The Bio-Traps® were sampled every month for 4 months. From each Bio-Trap®, 2.5 g beads were separated out and frozen until extraction. During the study period, no fine-grained or sand particles were observed in the Bio-Traps®. At the time of deployment (August 2013), the water column and sediment microbial communities were sampled at Fort Falls (surface location) and at both Bio-Trap® sample locations. Over the next 4 months at both Bio-Trap® locations, water column, surface sediment, and Bio-Trap® microbial communities were sampled. Only the water column and sediment microbial communities were sampled at the Fort Falls location at those times.

### DNA extraction and pyrosequencing

Total environmental nucleic acids were extracted from two Sterivex™ filters collected at each sampling location using a method modified from Riemann et al. (2008). Briefly, sucrose lysis buffer (0.75 M sucrose, 0.5 M Tris-HCl, 0.4 M EDTA) and 5 mg/mL lysozyme (Fisher BioReagents) were added to each filter prior to incubation at 37°C for 30 min. Proteinase K (100-μg/mL final concentration; Fisher BioReagents) and 10% SDS were added, and digestion continued at 55°C overnight. The lysate was drawn from the filter and combined with a 1X TE buffer wash of the filter prior to adding 0.3 M sodium acetate and molecular grade 100% isopropanol. Lysates were centrifuged and pellets were separated from the supernatants and resuspended in TE buffer. Nucleic acids were precipitated from the suspensions using 25:24:1 phenol:chloroform:isoamyl alcohol (pH 8) twice, and 24:1 chloroform:isoamyl alcohol once, prior to pelleting by centrifugation. Pellets were washed with 100% molecular grade ethanol twice and then resuspended in 1X TE buffer.

MoBio PowerSoil® Extraction kits, following manufacturer instructions (MoBio Laboratories, Inc., Carlsbad, CA, USA), were used to extract total nucleic acids from 0.25 g of beads collected from each Bio-Trap® and separately from 0.25 g of sediments at each sampling location. Extractions for each sample type per sample period and location were done in triplicate.

The quality and quantity of extracted DNA were verified by examining products on TBE agarose gels with ethidium bromide staining after electrophoresis and by measuring the ratio of absorbance maxima at 260 and 280 nm, and 260 and 230 nm, with a Thermo Scientific Nanodrop 2000c Spectrophotometer. Duplicate (for water) or triplicate (for Bio-sep® beads or sediment) extractions at a sampling location and month were homogenized prior to purification, barcoding, and amplicon pyrosequencing using a Roche 454 FLX Titanium instrument and reagents, as described in Dowd et al. (2008), at the Molecular Research LP (MrDNA) laboratory (www.mrdnalab.com; Shallowater, Texas, USA). The V1-V3 region of 16S rRNA genes was amplified using 27F-534R primers (Dowd et al., 2008).

### qPCR analyses

Bacterial biomass was estimated for all samples by quantitative PCR (qPCR) using a CFX96 Real-Time PCR System (Bio-Rad Laboratories, Hercules, CA, USA), according to the approach described by Ortiz et al. (2014). Briefly, for a 10-ml qPCR reaction with a 2x SensiFAST™ SYBR® No-ROX Kit (Bioline Meridian Life Science Company, Tauton, MA), 400 mg/mL bovine serum albumin solution, 400 nM of each primer, and 400 pg DNA extract were used. Primers used for bacterial 16S rRNA amplification were 338F and 518R (Ortiz et al., 2014). A standard curve was used to calculate the number of 16S rRNA amplicons (Zhu et al., 2005):

$$\begin{array}{r} \text{N}=\left[\left(A∕B\right)\times d\right]\times \left(\text{V}∕\text{C}\right) \end{array}$$

N is the total number of cells in the initial sample; *A* is the number of 16S rRNA amplicons per PCR tube, as calculated from the standard curve; *B* is the number of μL of cell lysate in the PCR tube, and *d* the lysate dilution factor; V is the initial lysate volume expressed in μL, and C is the average number of 16S rRNA copies per bacterial cell. Based on the retrieved bacterial diversity from our samples, and specifically of the predominance of Proteobacteria, we used the value 4.2 based on the genome assessment work of Vetrovsky and Baldrian (2013). N was divided by the amount of water filtered for each sample, or the amount of sediment or Bio-Trap® beads used during the extractions, to find the number of cells per mL of water, or the number of cells per gram of sediment or Bio-Traps®, respectively.

### Sequence analyses

Amplicon sequence data were quality screened and chimera checked prior to clustering into operational taxonomic units (OTUs) based on 95% sequence similarity using QIIME (Crawford et al., 2009; Caporaso et al., 2010; Edgar et al., 2011). A 95% cut-off was used to cluster OTUs at the genus level because of the short length of the pyrosequences (Kunin et al., 2010). The greengenes 13_8 database (Desantis et al., 2006) was used as the reference for the usearch61 method for chimera checking (Edgar et al., 2011) and for picking OTUs using the open reference method (Desantis et al., 2006). From the 18,177 OTUs generated for the full dataset (397,144 amplicons; Supplemental Table 1), representative sequences were chosen for classification by the RDP Classifier at 80% confidence intervals using QIIME (Wang et al., 2007). Alpha-diversity was calculated in QIIME to generate rarefaction curves (Supplemental Figure 3) (Crawford et al., 2009; Caporaso et al., 2010) and Shannon diversity (H') and Chao1 indices were calculated in the computer program R using the package phyloseq (version 1.10.0) (McMurdie and Holmes, 2013). Higher numbers for both indices indicate greater OTU-level richness. All OTUs shared between samples were compared for presence/absence. Details regarding data processing are provided in the Supplemental Materials and Methods in R markdown format.

All raw amplicons obtained from this study were submitted to the NCBI Sequence Read Archive (SRA) under the Bioproject PRJNA283038, with the accession numbers SAMN03451533–SAMN03451581 (http://www.ncbi.nlm.nih.gov). Summaries for the amplicon data, including SRA Accession Numbers for each sample, are included in Supplemental Table 1.

### Statistical analyses

The significance of changes in geochemical variables over time and between sampling months, as well as in microbial diversity data, were analyzed statistically using several approaches. Analysis of variance (ANOVA), reported as the *F*-test value with significance evaluated from a *p*-value of < 0.05, was done with geochemical data between month, season, and location using the car package in R (Fox and Weisberg, 2011). Summary code completed in R is included in the Supplemental Materials and Methods. Sediment grain size comparisons were done using the G2Sd package for sediment size analysis (Gallon and Fournier, 2013). Permutational multivariate analysis of variance (PERMANOVA), calculated with the Adonis function in the vegan package for R, was used to detect similarities in the means of multivariate groups described by material type (i.e., water, sediment, Bio-Trap®), location (i.e., surface, upstream, and downstream), and month, such that community OTU representation would be equivalent for all groups. PERMANOVA was also used to detect similarities in the composition and/or relative abundances of different OTUs based on geochemical variable (i.e., Cl, Ca, HIX, etc.). PERMANOVA was performed with the Adonis function from the vegan package for community ecology on a Bray-Curtis dissimilarity matrix and significance was assessed with 9999 permutations (Oksanen et al., 2013). Non-metric multidimensional scaling (NMDS) was used on a Bray-Curtis dissimilarity matrix to represent the pairwise dissimilarity graphically between OTUs in each sample. Statistically significant environmental variables (*p*-value < 0.05) were plotted as vectors representing the average of factor levels using envfit, from the vegan package (Oksanen et al., 2013).

To investigate any linear relationships between the distribution of OTUs among samples and any redundant geochemical gradients, a redundancy analysis (RDA) was performed. The significance of RDA axes was calculated by the PCAsignificance function in the BiodiversityR package (Kindt, 2014). To evaluate the relationship between OTU distribution among sediment samples and sediment size, another RDA was performed on only sediment samples. RDAs were performed with the RDA function from the vegan package on a Bray-Curtis dissimilarity matrix (Oksanen et al., 2013), which was produced using a culled dataset of only OTUs present more than three times in at least 20% of the samples (McMurdie and Holmes, 2013). Only 313 of the original 18,177 OTUs remained and application of a 2.0 CV cutoff resulted in 178 OTUs.

## Results

### Stream dynamics, sediment characteristics, and aqueous geochemistry

Several major rainfall events occurred during the study within the watershed (Supplemental Figure 2). Stream discharge fluctuated from unperceivable by flow meter to as high as 1.36 m^3^/s at the downstream location (Table 1). At these flow rates during the study period, sediment particles up to 2 mm in diameter may have been mobilized during four different precipitation events based on Stokes calculations. Excluding the largest particles (i.e., cobbles), coarse to very coarse sand (0.5–2 mm diameter) was sampled from the upstream location at Jones Cave. The average particle sizes downstream in the Lake Room were fine-medium sand (0.125–0.5 mm) (Supplemental Figure 4). There was <1% contribution of silt- or clay-sized particles at both sampling locations. After a large storm event in December (Supplemental Figure 2), sediment remobilization and redistribution was evident and finer particles were deposited at the downstream location (Supplemental Figure 4).

**Table 1:** **Geochemical and hydrological data from each sample**.

**Sample month**	**Sample location**	**Sample name**	**Temp °C**	**pH**	**Alkalinity mg/L**	**DOC^a^ mg/L**	**Total N^b^mg/L**	**Cl mg/L**	**SO^2−^_4_ mg/L**	**NO^−^_3_ mg/L**	**Na mg/L**	**Mg mg/L**	**Ca mg/L**	**Flow rate m/s^c^**	**Discharge m3/s^d^**	**FI^e^**	**HIX^f^**	**BIX^g^**
July	Surface	CCRW.13JB1	17.1	7.1	56.85	3.08	0.46	10.1	30.1	BDL	5.99	2.71	9.23	BDL	NC	1.97	6.97	0.63
July	Downstream	CCRW.13JA1	21.5	7.4	58.07	6.62	0.46	5.61	19.09	BDL	5.23	4.81	17.45	BDL	NC	2.0	10.33	0.58
July	Surface	CCRW.13JB3	21.5	7.5	84.66	NM	NM	25.58	33.34	BDL	12.24	10.25	8.34	0.78	0.21	2.01	11.08	0.61
July	Downstream	CCRW.13JA3	17.3	7.3	84.42	NM	NM	18.68	27.05	1.09	8.93	7.66	20.67	2.23	0.6	2.0	10.33	0.58
August	Surface	CCRW.13A3	24	7.2	84.42	3.0	0.77	17.84	31.03	1.41	8.11	8.50	10.22	5.25	0.82	2.01	8.51	0.65
August	Upstream	CCRW.13A2	20.3	7.3	93.6	5.2	0.99	11.6	24.31	1.28	7.74	6.89	14.78	4.4	0.69	1.99	8.29	0.64
August	Downstream	CCRW.13A1	18.3	7.3	87.84	NM	NM	10.59	17.01	1.03	6.69	5.56	15.3	6.23	1.68	1.96	8.88	0.64
September	Surface	CCRW.13SF3	22	7.4	107.36	3.0	0.31	24.23	28.62	0.27	12.53	10.46	41.48	BDL	NC	2.1	0.93	0.68
September	Upstream	CCRW.13S3	17.9	7.5	110.28	2.85	0.55	34.66	32.61	BDL	10.4	8.93	40.79	BDL	NC	2.14	0.93	0.66
September	Downstream	CCRW.13SL3	18	7.4	127.36	1.79	0.65	19.07	21.69	1.55	9.52	7.39	47.09	BDL	NC	2.11	0.91	0.66
October	Surface	CCRW.13O3	16.9	7.7	125.41	0.27	0.28	30.12	29.37	BDL	11.34	10.24	38.62	BDL	NC	2.11	0.92	0.69
October	Upstream	CCRW.13O2	15.2	7.7	152.01	2.54	0.33	23.78	30.94	BDL	10.22	10.19	41.5	BDL	NC	2.12	0.93	0.69
October	Downstream	CCRW.13O1	13.7	7.8	133.95	3.81	0.57	22.59	20.37	1.05	7.64	7.05	46.21	BDL	NC	2.12	0.93	0.69
November	Surface	CCRW.13N3	10.1	7.5	105.65	1.48	0.32	23.49	38.44	BDL	11.73	9.93	32.02	0.76	0.2	2.13	0.91	0.67
November	Upstream	CCRW.13N2	8.7	7.5	88.57	2.48	0.40	20.09	34.61	BDL	9.68	9.16	35.66	3.2	0.05	2.19	0.91	0.69
November	Downstream	CCRW.13N1	10	7.5	130.05	3.99	0.42	22.28	29.44	0.82	9.64	7.73	40.22	1.67	0.14	2.16	0.9	0.7
December	Surface	CCRW.13D3	4.3	7.1	93.2	1.7	0.93	17.9	35.23	BDL	9.44	6.09	9.00	3.7	10.18	2.04	0.9	0.59
December	Upstream	CCRW.13D2	3.8	7.3	62.46	1.67	0.98	19.45	33.77	BDL	14.59	7.35	13.16	4.95	0.7	2.12	0.9	0.66
December	Downstream	CCRW.13D1	4.9	7.5	93.2	1.69	0.56	18.33	28.32	BDL	9.29	6.52	15.63	2.95	1.32	2.2	0.9	0.66

NM, not measured.

a DOC, dissolved organic carbon measured as the difference between dissolved non-purgable organic carbon and total inorganic carbon.

b Total N, total dissolved nitrogen measured as all N compounds present in sample, including nitrogen compounds in DOM.

c Water flow rate; BDL, below detection limit for the flow meter measurement.

d NC, not calculated because velocity measurements were below detection.

e FI, Fluorescence index, see text for description.

f HIX, Humification index, see text for description.

g BIX, Biological index, see text for description.

Geochemical parameters for all of the stream water pH, ranging from 7.1 to 7.8, at each location significantly varied by month (ANOVA *F*-test = 33; *p*-value < 0.001), as did stream temperature, ranging from 21°C (July) to 4°C (December) (*p*-value < 0.001) (Table 1). Other geochemical parameters, including alkalinity, also significantly differed by month (ANOVA *F*-test = 8.7; *p*-values for all analyses < 0.05). The amount of DOC (ranging from 0.27 to 6.6 mg/L) and total dissolved N (ranging from 0.33 to 1 mg/L) did not significantly differ for any analysis by month or between locations. However, the quality of the carbon, as assessed by using fluorescence indices FI and HIX (Table 1), did significantly differ by month (Supplemental Table 2). In July and August, CDOM fluorescence was dominated by humic acids derived from terrigenous material and less proteinaceous CDOM than later months in the Fall and Winter seasons.

### Controls on bacterial biomass and diversity

**Figure 2:**
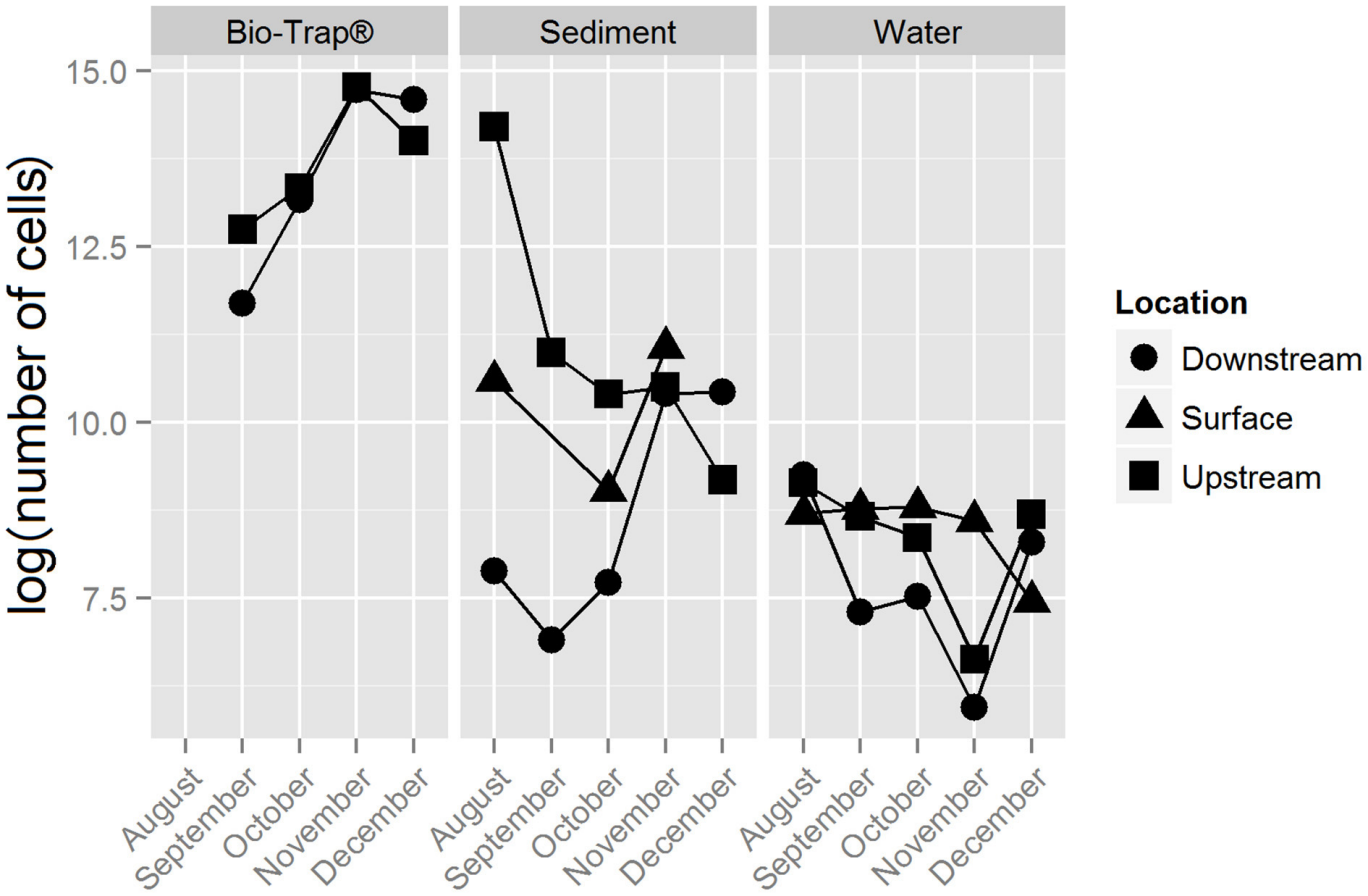
**Bio-Trap®, sediment, and water biomass estimates from qPCR results, displayed as log (number of cells) over time for each type of sample at the surface, upstream, and downstream locations**.

The number of 16S rRNA gene copies qPCR reaction ranged from 1 × 10^5^ to 1 × 10^2^ copies/sample, which was used to calculate biomass per gram of sediment or Bio-Trap® beads, or per mL water. Bio-Trap® samples had higher biomass (up to 2.6 × 10^6^ cells/gram) than the other sample types; water had the least biomass at only 1 × 10^4^ cells/mL (Figure 2). Sediment biomass was greatest in August and decreased through the winter months, but biomass in the cave stream was relatively stable throughout the study period. Biomass in the Bio-Trap® samplers for both sampling locations were nearly the same, with the least biomass at the beginning of the experiment and the highest biomass in November.

**Figure 3:**
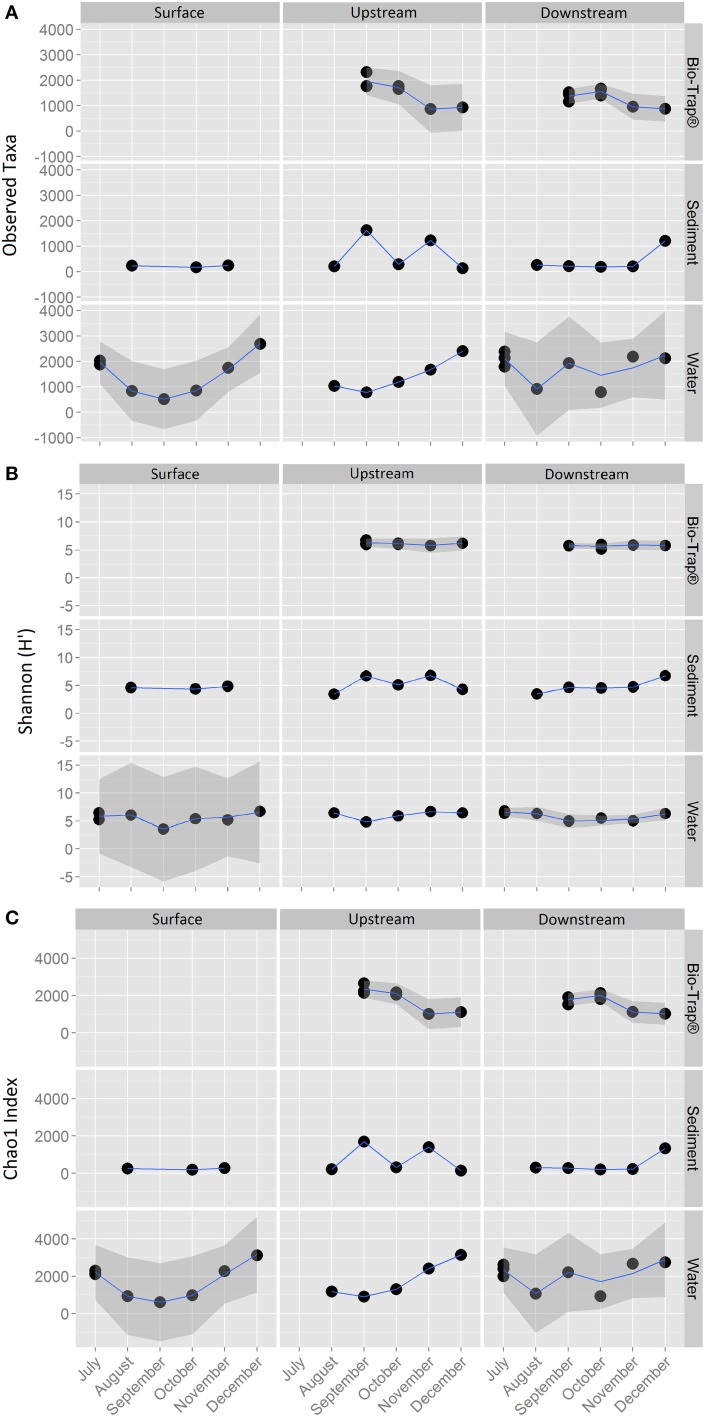
**Alpha-diversity richness and evenness indices for (A) Observed, (B) Shannon, and (C) Chao1, values by sample type and location over a 6 month period**.

The 18,177 OTUs were affiliated with 402 classified genera. The most abundant classes for all the OTUs included Betaproteobacteria (35% of all sequences), Gammaproteobacteria (16% of all sequences), Alphaproteobacteria (15% of all sequences), and Opitutae (4% of all sequences). The planktonic community throughout the cave stream was dominated by Betaproteobacteria (48%), Alphaproteobacteria (8%), and Opitutae (6%). The sediment samples throughout the cave were dominated by Gammaproteobacteria (34%), followed by Alphaproteobacteria (16%) and Betaproteobacteria (12%). The Bio-Trap® communities from both locations had nearly equal distributions of Betaproteobacteria (26%), Alphaproteobacteria (24%), and Gammaproteobacteria (23%). Over time, observed Bio-trap® community OTU abundances decreased (Figure 3A), but calculated richness and evenness were unchanged (according to H' and Chao1, Figures 3B,C, respectively).

**Figure 4:**
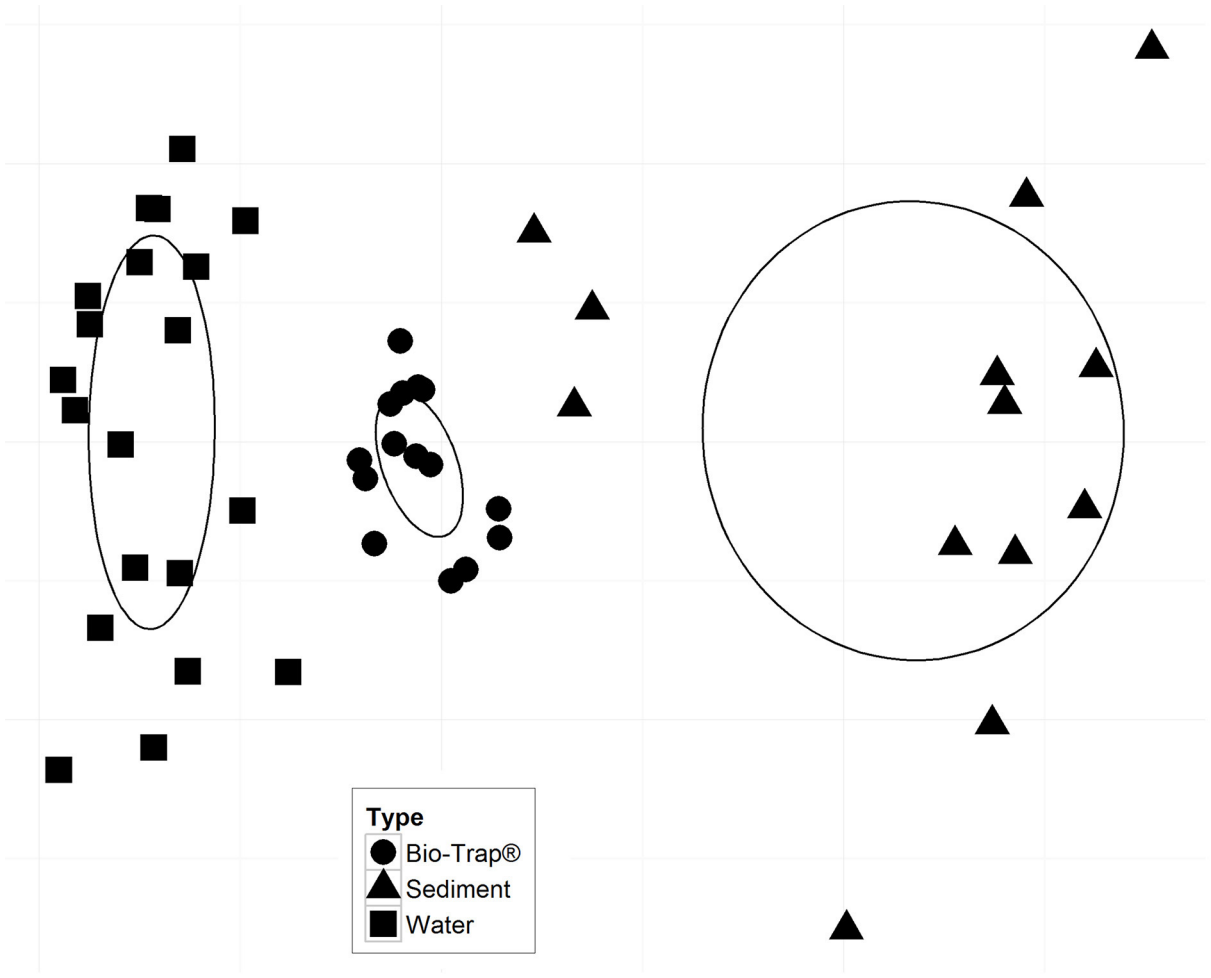
**Non-metric multidimensional scaling (NMDS) plot based on a Bray-Curtis dissimilarity matrix; stress = 0.082**. Ellipses represent the standard error of the weighted average of scores of samples, and the direction of the principal axis of the ellipse is defined by the weighted correlation of samples. There were no statistically significant environmental vectors (*p*-value < 0.05).

**Figure 5:**
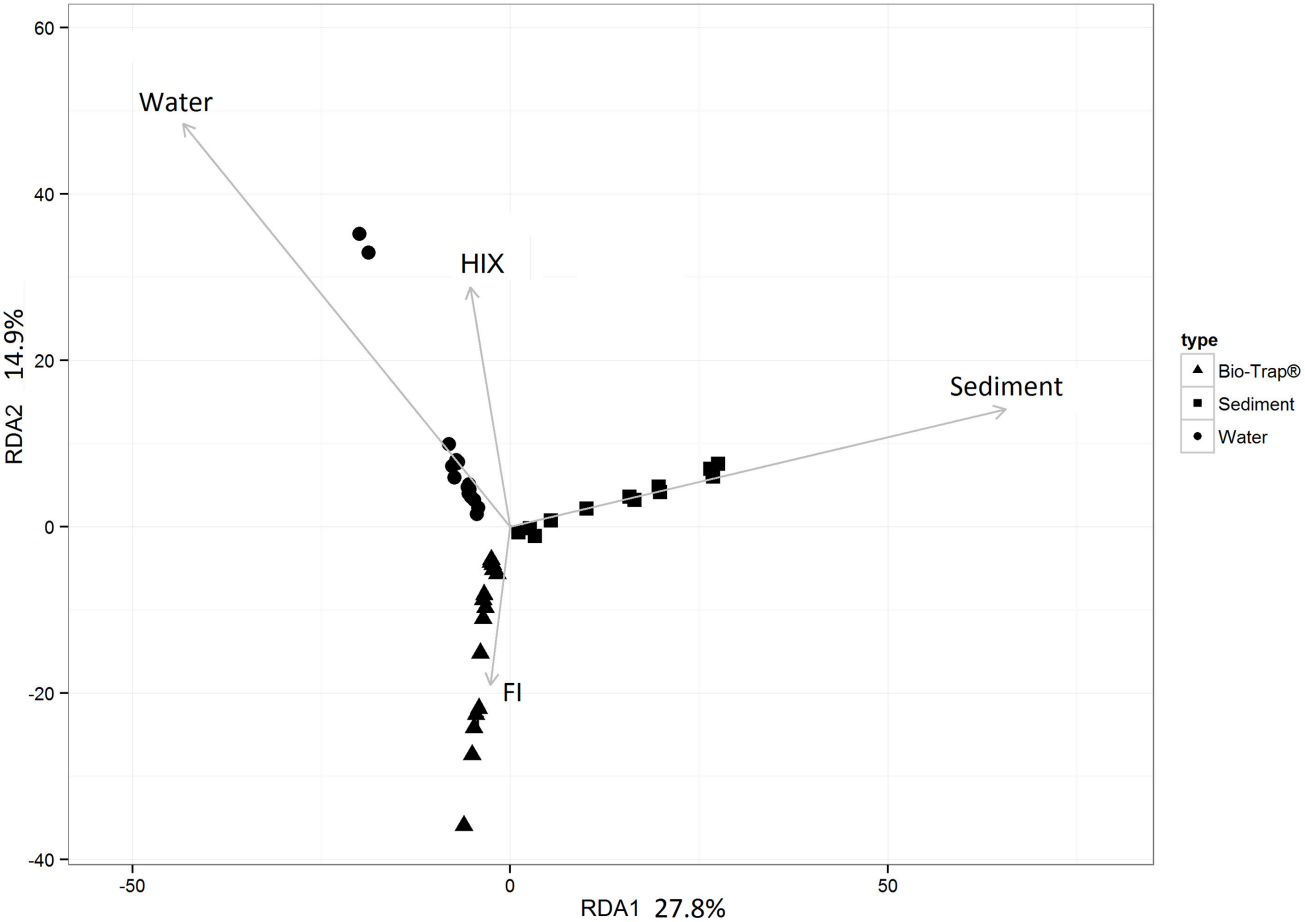
**Redundancy analysis (RDA) of the culled OTU dataset as a function of the fluorescence indices HIX and FI**. Significance of each RDA axis was calculated with the RDAsignificance function from the BiodiversityR package for R (Kindt, 2014).

**Figure 6:**
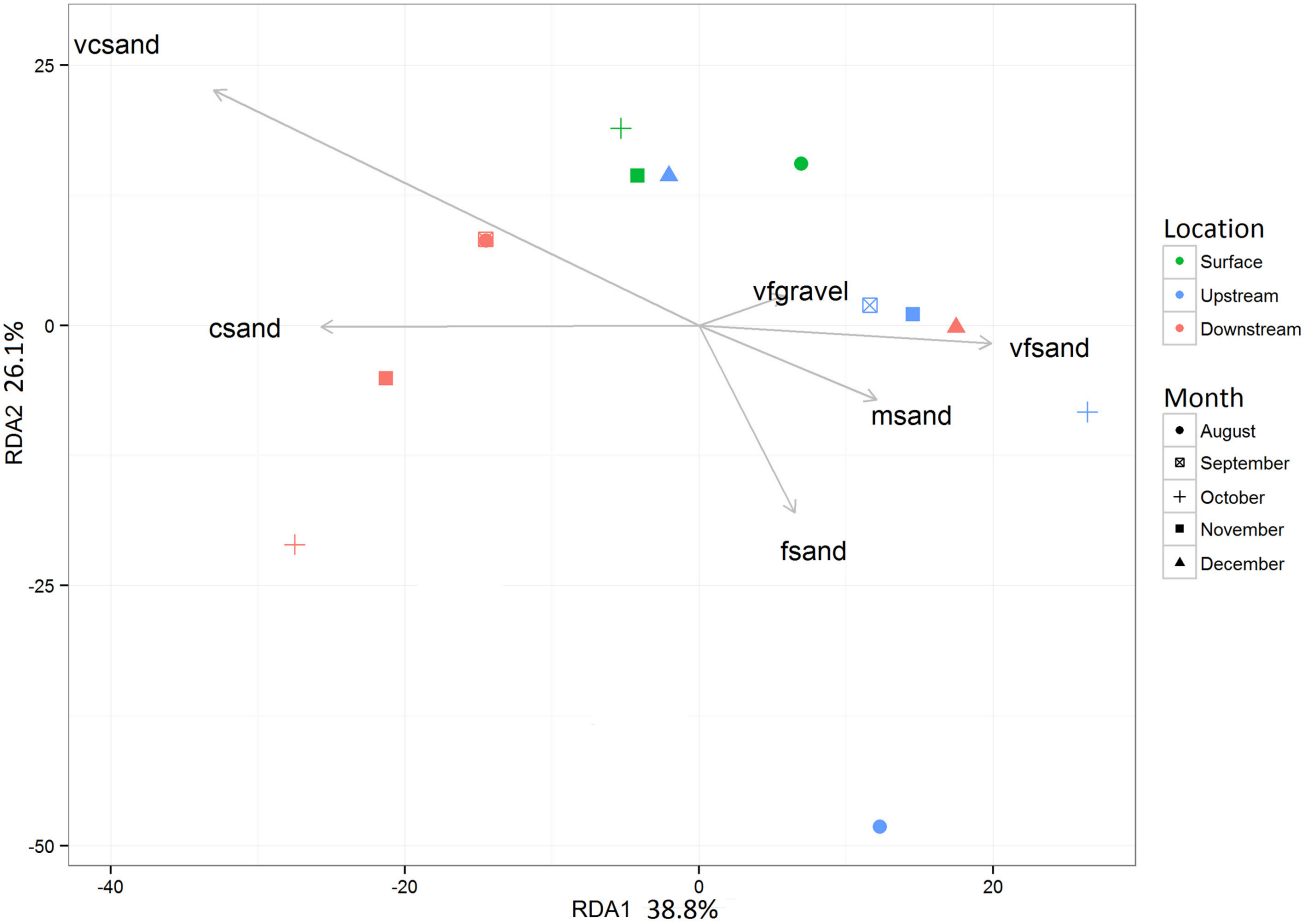
**Redundancy analysis (RDA) of the culled OTU dataset as a function of the grain size analysis from the G2SD package gran_stat function output (Gallon and Fournier, 2013)**. Significance of each RDA axis was calculated with the RDAsignificance function from the BiodiversityR package for R (Kindt, 2014).

Prior to testing hypotheses related to AMEC existence and community succession, changes in bacterial diversity based on environmental gradients over time were evaluated. Each sample's taxonomic profile was compared temporally and spatially. Overall OTU taxonomic distribution between locations was significantly distinct from each other (i.e., upstream vs. downstream) (PERMANOVA *p*-value < 0.05, *r*^2^ = 6%), and taxonomy differed significantly by month (PERMANOVA *p*-value < 0.001, *r*^2^ = 18%). OTU taxonomy clustered significantly by sample type (i.e., water, sediment, Bio-Traps®), according to both ordination in NMDS space (Figure 4) and a RDA (Figure 5) that tested potential multidimensional and linear relationships among environment gradients and taxonomy, respectively. Changes in seasonal CDOM quality from FI and HIX fluorescence indices accounted for observed bacterial diversity variation for water and Bio-Trap® samples, but not the sediment samples (RDA axis 2, 14.9%; Figure 5). Instead, diversity from the sediment samples clustered by location and according to sediment size (Figure 6), which also differed over time.

### Shared community membership and potential succession

**Figure 7:**
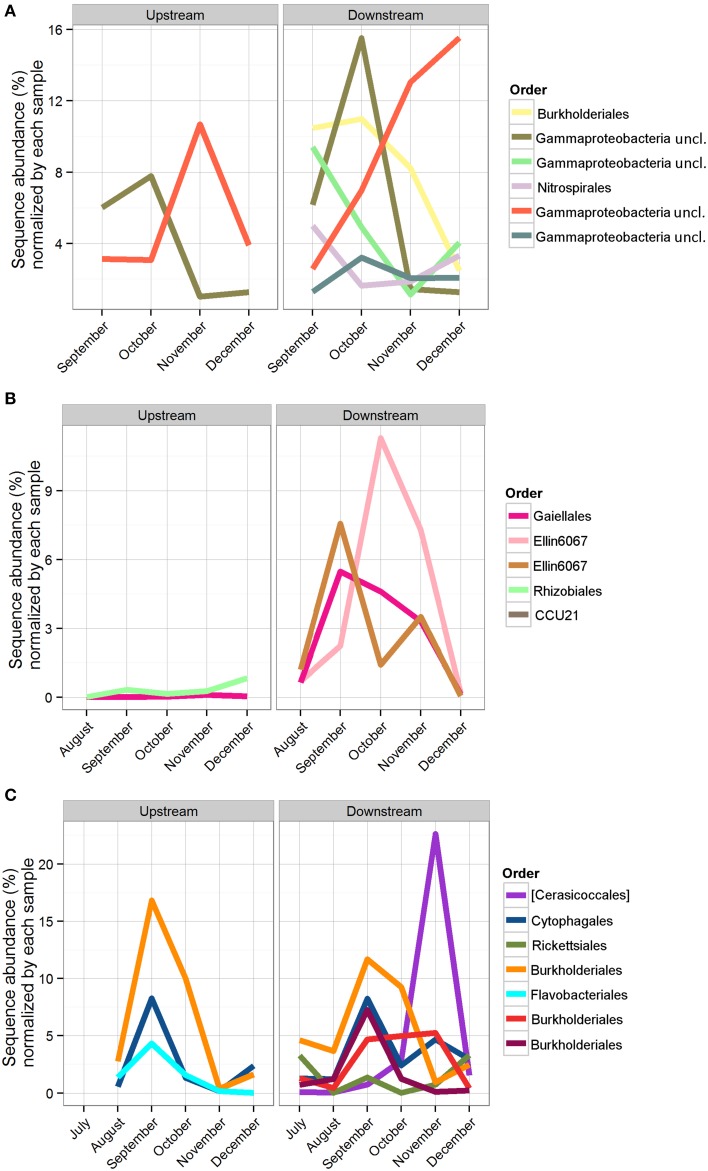
**Sequence abundance of OTUs present for the duration of the study, normalized by the total abundance of sequences in the sample**. Each OTU is colored by its taxonomic order, and the same color represents the same OTU across locations. **(A)** Bio-Trap® samples, triplicates were averaged for the sequence abundances; **(B)** Sediment samples; **(C)** Water samples.

The number of shared OTUs was evaluated based on sample location, type (sediment, water, Bio-Traps®), and month to assess community stability, which could potentially provide evidence for AMEC. A shared OTU was identified if amplicons from more than one sample type, location, or month were present. Overall, the number of shared OTUs for any location or sample month was low, between 0.1 and 4% (Table 2), in contrast to the total number of OTUs retrieved during the 6 months. No OTUs comprised amplicons from all sediment, water, and Bio-Trap® samples from any location and any month (Table 2). But, there were shared OTUs from the sediments, water, and Bio-Traps® at each location over the 6 month study period (Table 3; Figure 7), although the total number of shared OTUs was different for each material. Specifically, shared OTUs for sediment samples were comparatively lower (0.01–4% of the total) than the water and Bio-Trap® samples, which shared 20–65% of the OTUs when binned by sample type. The shared and prevalent OTUs over time showed sequence abundance changes (Figure 7). Some of the most prevalent OTUs had a similar trend over time in both upstream and downstream locations (Figure 7).

**Table 2:** **Number of shared OTUs by taxonomic Phylum and Class for each sample type upstream and downstream, as well as shared among both locations (represented by the “shared” column), over time**.

**Phylum**	**Class**	**Bio-Trap®**			**Sediment**				**Water**			
		**Upstream**	**Downstream**	**Shared**	**Surface**	**Upstream**	**Downstream**	**Shared**	**Surface**	**Up-stream**	**Down-stream**	**Shared**
Acidobacteria	Acidobacteria-6	8 (1.3)	4 (1.1)	4 (1.3)	1 (4.2)	1 (0.2)	1 (0.4)	−	−	−	−	−
Acidobacteria	[Chloracidobacteria]	3 (0.2)	1 (0)	1 (0.1)	−	−	−	−	−	−	−	−
Actinobacteria	Acidimicrobiia	−	−	−	2 (2.8)	−	−	−	−	−	−	−
Actinobacteria	Thermoleophilia	−	−	−	5 (3.8)	−	3 (4.1)	−	−	−	−	−
Actinobacteria	Actinobacteria	−	−	−	−	−	−	−	1 (0.1)	3 (1.1)	4 (1.6)	2 (0.4)
Chloroflexi	Ellin6529	−	−	−	1 (1.3)	−	1 (0.2)	−	−	−	−	−
Chloroflexi	P2-11E	−	−	−	2 (0.5)	−	−	−	−	−	−	−
Chloroflexi	Anaerolineae	3 (0.3)	−	−	−	−	−	−	−	−	−	−
Chloroflexi	Chloroflexi	1 (0)	−	−	−	−	−	−	−	−	−	−
Proteobacteria	Alphaproteobacteria	88 (14.9)	61 (12.8)	48 (12.0)	3 (3.1)	−	8 (5)	−	−	9 (1.3)	2 (2)	2 (1.7)
Proteobacteria	Betaproteobacteria	52 (12.8)	55 (14.2)	38 (13.3)	4 (1.6)	1 (0)	3 (2.6)	−	53 (20.2)	39 (14.6)	41 (18.2)	19 (13.1)
Proteobacteria	Deltaproteobacteria	1 (0)	1 (0)	11 (17.3)	−	−	−	−	−	−	−	−
Proteobacteria	Gammaproteobacteria	18 (16.6)	24 (18.6)	−	−	−	−	−	−	3 (0.3)	2 (0)	−
Proteobacteria	Epsilonproteobacteria	−	−	−	−	−	−	−	1 (0.2)	−	−	−
Proteobacteria	NA	−	2 (0.8)	−	−	−	−	−	−	−	−	−
[Thermi]	Deinococci	1 (0)	−	−	−	−	−	−	−	−	−	−
Bacteroidetes	[Saprospirae]	3 (0.6)	5 (0.7)	1 (0.5)	−	−	−	−	−	−	1 (0.1)	−
Bacteroidetes	Cytophagia	5 (0.9)	7 (1.5)	5 (1.3)	−	−	−	−	1 (1.3)	4 (3.2)	2 (4)	1 (3.6)
Bacteroidetes	Sphingobacteriia	1 (0)	4 (0.4)	−	−	−	−	−	−	−	−	1 (1.0)
Bacteroidetes	Flavobacteriia	−	−	−	−	−	−	−	1 (0.7)	4 (1.5)	1 (0.8)	−
Gemmatimonadetes	Gemmatimonadetes	1 (0)	2 (0.1)	−	−	−	−	−	−	−	−	−
Nitrospirae	Nitrospira	5 (2.6)	6 (8.6)	5 (6.4)	−	−	−	−	−	1 (0.1)	−	−
Planctomycetes	OM190	1 (0)	1 (0)	−	−	−	−	−	−	−	−	−
Planctomycetes	Planctomycetia	9 (0.9)	6 (1.1)	1 (0.7)	−	−	−	−	−	−	−	−
Planctomycetes	vadinHA49	−	−	−	−	−	−	−	1 (0)	−	−	−
Verrucomicrobia	Opitutae	2 (0.2)	3 (0.3)	2 (0.3)	−	−	−	−	1 (0)	7 (0.6)	3 (5.1)	2 (0.2)
Armatimonadetes	Armatimonadia	−	1 (0)	−	−	−	−	−	−	−	−	−
Elusimicrobia	Elusimicrobia	−	1 (0)	−	−	−	−	−	−	−	−	−
Cyanobacteria	Chloroplast	−	−	−	−	−	−	−	2 (0.6)	−	−	−
OP3	PBS-25	−	−	−	−	−	−	−	1 (0.1)	1 (0.1)	−	−
Fibrobacteres	Fibrobacteria	−	−	−	−	−	−	−	−	1 (0)	−	−
NA	NA	1 (0)	−	−	−	−	−	−	−	−	−	−
Totals												
#OTUs shared in group		203 (4)	184 (4.2)	116 (2.2)	18 (3.4)	2 (0.1)	16 (0.9)	−	62 (1)	72 (1.5)	56 (0.7)	27 (0.3)
# OTUs in group		5107	4342	5107	528	2527	1697	−	6495	4875	8025	8025
# Of sequences in group		63683	76729	140412	6029	34942	13172	−	74936	17472	35245	127653

Numbers in parentheses represent the percent abundance, determined to be the number of sequences in shared OTUs normalized by the shared group total.

**Table 3:** **Number of OTUs shared between Bio-Trap® samples and Water/Sediment environment types in both August and December for both locations inside the cave**.

	**Bio-Trap®**			
	**Upstream August**	**Upstream December**	**Downstream August**	**Downstream December**
Water	153	216	110	199
Sediment	12	26	13	212

To assess community succession, comparisons among shared OTUs from sediment, water, and Bio-Traps® were made. Evidence for community succession was indicated if OTUs were comprised of amplicons from Bio-Traps® and either water or sediment over time. Upstream and downstream Bio-Trap® samples had more shared OTUs with water (20 OTUs upstream and 13 downstream) than with sediment (0 OTUs upstream and 1 downstream). Downstream, the number of shared OTUs between Bio-Traps® and sediments increased by the end of the study (Table 3). This trend was not observed upstream, as the number of shared OTUs between Bio-Traps® and sediments remained low (Table 3).

## Discussion

Originally described from karst spring water, AMEC represent stable communities that develop over months to years and that form from a mix of planktonic and biofilm (i.e., attached) communities within a karst aquifer (Farnleitner et al., 2005; Pronk et al., 2008). Karst aquifers have interconnected networks of solutionally-enlarged conduits and voids, solutionally-enlarged fractures and bedding partings, and bedrock matrix. Each component has its own flow regime, ranging from fast and potentially turbulent flow in conduits to Darcian or diffusive flow in fractures and the matrix (Ford and Williams, 2013). AMEC have previously been found within saturated conduits and voids and along fractures in the subsurface, where flow may be fast but residence times are long so environmental conditions remain stable, particularly pH and temperature (Farnleitner et al., 2005; Pronk et al., 2008). When AMEC were originally described, attached communities were not analyzed, presumably due to difficulties sampling karst bedrock surfaces from wells (Engel and Northup, 2008). Well boreholes completed in karst aquifers usually intercept fractures, conduits, and voids, and nothing but water can usually be physically sampled when voids are encountered. Moreover, these zones are cased off during well completion and inhibit future access to aquifer bedrock surfaces. Karst well construction and sampling contrasts other groundwater systems, such as porous sand and gravel aquifers, because aquifer sediment and/or rock material can be physically sampled from cores during well construction. From these other types of groundwater systems, planktonic and attached microbial communities can be distinct based on taxonomic (Hazen et al., 1991; Alfreider et al., 1997; Lehman, 2007; Flynn et al., 2008; Zhou et al., 2012) and functional diversity (Wilhartitz et al., 2009). Moreover, planktonic microbial communities in porous sand and gravel aquifers can be seasonally dynamic while sediment-attached communities are unchanging (Zhou et al., 2012). Understanding how AMEC form and evolve is important because karst systems are highly susceptible to contamination (Vesper et al., 2001) and AMEC may play an important role the stability of microbial communities during ecosystem biogeochemical cycling or contaminant response.

Caves allow for direct entry into karst aquifer systems (Yagi et al., 2010; Morasch, 2013). Prior to this study, knowledge about cave stream bacterial diversity was limited and understanding how environmental parameters impact cave stream bacteria was poor (Engel, 2010). The hydrology of cave streams is different from that of the original AMEC habitats because residence times can be much shorter, on the order of hours to days, and environmental conditions can vary daily (Farnleitner et al., 2005; Pronk et al., 2008). Cave streams are hydrologically comparable to surface streams, and stable communities comparable to AMEC have not yet been identified from surface streams (Lyautey et al., 2005; Besemer et al., 2007, 2012; Lear et al., 2008; Wey et al., 2012). However, in surface streams, sediment-attached microbial communities have been shown to express seasonal diversity trends (Feris et al., 2003; Hullar et al., 2006; Wey et al., 2012) and the distribution of planktonic bacteria and bacteria attached to fine benthic organic matter also correlates to surface stream pH (Fierer et al., 2007). As such, because cave streams are hydrologically connected to the surface, seasonal trends linked to physicochemistry may be observed from cave stream microbial communities. We found that, although there were significant differences for some environmental parameters over time, there were no significant differences in bacterial diversity over time at any one location along the cave stream. The duration of study may have been too short to observe potential lasting effects of seasonality on community assembly.

Conceptually, there is a low probability of AMEC development in cave streams because of more rapid removal or redistribution of material of all sizes (from clay particles to large logs), including microbial communities. In contrast to the original AMEC studies of planktonic communities (Farnleitner et al., 2005; Pronk et al., 2008), we hypothesized that sediment communities would be compositionally stable over time and provide evidence for AMEC formation because planktonic communities would likely be dominated by transient populations from the surface and stream water residence times would be too short for autochthonous communities to develop, in contrast to cave pools (Shabarova and Pernthaler, 2010; Shabarova et al., 2013, 2014). There were shared OTUs among the water samples throughout the entire study (Table 2), and the shared OTUs between the surface water and cave water indicated that some of the planktonic bacteria were ubiquitously distributed throughout the cave system (Table 2). This may be due to their survival throughout the duration of the flowpath, not that they are AMEC. In prior studies, to indicate a unique habitat consistent with microorganisms sourced autochthonously from within a system, >30% of total sequences should be considered unclassified (<50% sequence similarity) past the domain level (Farnleitner et al., 2005; Pronk et al., 2008). From alpine systems, AMEC consist of Acidobacteria, Nitrospira, Gammaproteobacteria, and Deltaproteobacteria (Farnleitner et al., 2005; Pronk et al., 2008). In our study, the diversity of shared OTUs from the cave stream was different than previously described AMEC. Compared to the full bacterial diversity, the shared communities represented very little of the total diversity retrieved for all sample types (<4%; Table 2). Consequently, we do not believe there is sufficient evidence that AMEC developed in the cave stream water. Also, as was originally described, AMEC should represent common bacterial groups that occur both in the water and from attached biofilms on sediments and aquifer surfaces (Farnleitner et al., 2005; Pronk et al., 2008). Sediment remobilization would cause similarity in planktonic and sediment-attached communities. However, our results do not support this because there were few OTUs shared between water and sediment communities over time (Table 2). But, as separate habitats, water and sediments each shared OTUs throughout the entire study period (Table 2). Sediments at each location had distinct bacterial community compositions (Figure 5) that correlated to sediment size. At the upstream location, only two OTUs were shared (representing 0.1% of the overall community) over time, perhaps because sediment upstream may be more transient than downstream. Downstream, 16 OTUs, or 0.9% of the total diversity, were shared over time, and were comprised of Alpha- and Betaproteobacteria, Chloroflexi, Actinobacteria, and Acidobacteria. There were no OTUs shared between the surface sediments and upstream cave sediments, but four OTUs were shared between the surface sediments and downstream cave sediments. This may provide evidence that the cave sediment communities are not endemic to the karst system, but more work needs to be done in the future and over longer periods of time to verify this result.

One reason why there is limited evidence for AMEC in the cave stream may be linked to the frequency of flooding. Significant rainfall events have the capacity to mobilize sediments of certain sizes. Based on calculated volume estimates for the different areas of the cave, flooding frequency, and particle size distribution, the smaller sediment upstream in the cave was probably only in place at most 8 weeks during the study period. For AMEC to form in cave sediments, we would expect that the sediments should remain in place, or that attached communities are able to colonize newly (re)deposited sediments after an extended period of time. This would also increase the ability to readily distinguish AMEC from transient microbial communities. The monthly sampling intervals during the study period may have been too long to capture a stable community in the sediments because AMEC diversity was not easily distinguished from the sediments. Collectively from these results, it is unclear that AMEC, as defined originally (Farnleitner et al., 2005), formed in the cave stream sediments that were sampled in this study. We should point out that our sampling was biased toward smaller sediment sizes, and AMEC may develop on larger cobbles and boulders that are not mobilized as frequently as the smaller sediment sizes. Future work should sample the large sediment particles and the submerged cave wall and stream bottom surfaces because AMEC may be present on more stable surfaces in the stream.

Lastly, we examined the potential for successional patterns in cave stream communities by using artificial substrates (i.e., Bio-Trap® samplers). Knowledge about community succession in cave stream systems has been completely lacking. We hypothesized that Bio-Trap® communities would resemble sediment communities over time and we compared the community compositions among the planktonic and sediment-attached communities with those of the Bio-Traps®. Initially, even though the upstream and downstream planktonic communities differed, the Bio-Traps® at the upstream and downstream locations were dominated by OTUs shared with water at each location. Differences between the upstream and downstream communities were likely due to stochastic effects and dispersal potential (Fierer et al., 2010), but it is clear from the data that the planktonic microorganisms were the pioneering community for the Bio-Traps®. From a succession perspective, the downstream Bio-Traps® had more OTUs comprised of sediment amplicons at the end of the study (Table 3), but the upstream Bio-Traps® had the same small number of sediment-shared amplicons throughout the study. These results imply that the rate at which sediment-attached microorganisms colonize new surfaces differs depending on the location along the cave stream flowpath. At the end of the study, the relative abundances of several shared OTUs decreased at the upstream location but increased at the downstream location, suggesting that distinct Bio-Trap® communities formed according to the environmental conditions at each location (Fierer et al., 2010).

Variance among Bio-Trap® and water bacterial community compositions was positively correlated with CDOM quality along the cave stream flowpath, but CDOM quality did not correlate to sediment microbial community diversity. Bio-Trap® communities were likely utilizing CDOM in the water and not the sediments. This distinction is consistent with surface stream studies (Hullar et al., 2006), as well as karst aquifers (Simon et al., 2010), and the differences may be due to organic matter in the streambed being partitioned differently from the water column (Simon et al., 2010). Although the effect of temperature on the nature of CDOM in surface streams has been shown to play an important role in planktonic bacterial community structure and function (Van Der Gucht et al., 2005; Hullar et al., 2006), it is still unclear how environmental conditions affect CDOM in the cave streams and subsequent microbial community composition and assembly. Cave streams lack CDOM photodegradation, as well as the active photosynthesis that occurs in surface streams, which means that CDOM transported into the cave from the surface has the potential to retain its original properties. But, as CDOM is cycled along the flowpath, upstream CDOM is transformed and transported downstream or into the sediments for additional processing. The potential for CDOM quality to diminish with increasing travel time downstream may impact the composition and assembly of heterotrophic communities along the flowpath. The type of heterotrophic community that developed in the cave stream over time is consistent with exogenous (vs. endogenous) communities because these commonly form aquatic biofilms under reduced light conditions and reach a diversity plateau with only small shifts in biomass once the community reaches the plateau phase (Fierer et al., 2010). This is observed from the Bio-Trap® samplers with a biomass peak in November (Figure 3) (Fierer et al., 2010). Future research should address if specific differences exist regarding the nature and behavior of water vs. sediment organic matter and how those changes affect exogenous community composition and assembly over time.

In conclusion, microbes are essential for organic carbon and nutrient cycling in karst systems (Gibert et al., 1994; Simon et al., 2007). We found several distinct shared planktonic and attached bacterial communities in the cave stream, which is a novel outcome. However, although we found shared OTUs that were stable for the duration of our study, there were no OTUs shared between the planktonic and attached microbial communities. Therefore, we found limited evidence for AMEC in this cave stream. Nevertheless, the definition of AMEC should be updated, as we struggled during our data analysis to find a set of ubiquitous requirements that could be used for comparison. The Bio-Trap® bacterial communities that stabilized over time in both upstream and downstream locations along a cave stream provide evidence that succession following a large-scale (perhaps sterilizing) environmental disturbance does occur in cave streams (Fierer et al., 2010). Despite the many flooding events during this study period, the community richness trend was predictable over time for all the Bio-Trap® samples, even though the pioneering microbial community was not the same. Sediment size and mobilization play key roles in the sediment-attached karst microbial community structure. Organic carbon quality governs the planktonic karst microbial community structure in a cave stream. These findings also indicate that cave stream communities with short water residence times can follow successional patterns in response to disturbances, like flooding or contamination events, although community stability only exists for short periods of time between disturbances.

### Conflict of interest statement

The authors declare that the research was conducted in the absence of any commercial or financial relationships that could be construed as a potential conflict of interest.

## References

[B1] Alfreider A. Krossbacher M. Psenner R. (1997). Groundwater samples do not reflect bacterial densities and activity in subsurface systems. Water Res. 31, 832–840.

[B71] American Public Health A. Eaton A. D. American Water Works A. Water Environment F. (2005). Standard Methods for the Examination of Water and Wastewater. Washington, DC: APHA-AWWA-WEF.

[B2] ASTM (2008). D5373-08. Standard test methods for instrumental determination of carbon, hydrogen, and nitrogen in laboratory samples of coal, in Annual Book of ASTM Standards (West Conshohocken, PA), 19428–12959.

[B3] Besemer K. Peter H. Logue J. B. Langenheder S. Lindstrom E. S. Tranvik L. J.. (2012). Unraveling assembly of stream biofilm communities. ISME J. 6, 1459–1468. 10.1038/ismej.2011.205 22237539 PMC3400417

[B4] Besemer K. Singer G. Limberger R. Chlup A. K. Hochedlinger G. Hodl I.. (2007). Biophysical controls on community succession in stream biofilms. Appl. Environ. Microbiol. 73, 4966–4974. 10.1128/AEM.00588-07 17557861 PMC1951047

[B5] Birdwell J. E. Engel A. S. (2010). Characterization of dissolved organic matter in cave and spring waters using UV–Vis absorbance and fluorescence spectroscopy. Org. Geochem. 41, 270–280. 10.1016/j.orggeochem.2009.11.002

[B6] Bonacci O. Pipan T. Culver D. C. (2008). A framework for karst ecohydrology. Environ. Geol. 56, 891–900. 10.1007/s00254-008-1189-0

[B7] Caporaso J. G. Kuczynski J. Stombaugh J. Bittinger K. Bushman F. D. Costello E. K.. (2010). QIIME allows analysis of high-throughput community sequencing data. Nat. Methods 7, 335–336. 10.1038/nmeth.f.303 20383131 PMC3156573

[B8] Chapelle F. H. (2000). The significance of microbial processes in hydrogeology and geochemistry. Hydrogeol. J. 8, 41–46. 10.1007/PL00010973

[B9] Coble P. (1996). Characterization of marine and terrestrial DOM in seawater using excitation-emission spectroscopy. Mar. Chem. 51, 325–346.

[B10] Crawford P. A. Crowley J. R. Sambandam N. Muegge B. D. Costello E. K. Hamady M.. (2009). Regulation of myocardial ketone body metabolism by the gut microbiota during nutrient deprivation. Proc. Natl. Acad. Sci. U.S.A. 106, 11276–11281. 10.1073/pnas.0902366106 19549860 PMC2700149

[B11] Desantis T. Z. Hugenholtz P. Larsen N. Rojas M. Brodie E. L. Keller K.. (2006). Greengenes, a chimera-checked 16S rRNA gene database and workbench compatible with ARB. Appl. Environ. Microbiol. 72, 5069–5072. 10.1128/AEM.03006-05 16820507 PMC1489311

[B12] Dogwiler T. Wicks C. M. (2004). Sediment entrainment and transport in fluviokarst systems. J. Hydrol. 295, 163–172. 10.1016/j.jhydrol.2004.03.002

[B13] Dougherty P. H. (1985). An overview of the geology and physical geography of Kentucky, in Caves and Karst of Kentucky, ed Percy H. (Dougherty, KY: Geol. Survey Special Publication XI), 79–80.

[B14] Dowd S. E. Callaway T. R. Wolcott R. D. Sun Y. McKeehan T. Hagevoort R. G.. (2008). Evaluation of the bacterial diversity in the feces of cattle using 16S rDNA bacterial tag-encoded FLX amplicon pyrosequencing (bTEFAP). BMC Microbiol. 8:125. 10.1186/1471-2180-8-125 18652685 PMC2515157

[B15] Edgar R. C. Haas B. J. Clemente J. C. Quince C. Knight R. (2011). UCHIME improves sensitivity and speed of chimera detection. Bioinformatics 27, 2194–2200. 10.1093/bioinformatics/btr381 21700674 PMC3150044

[B16] Engel A. S. (2010). Microbial diversity of cave ecosystems, in Geomicrobiology: Molecular and Environmental Perspective, eds Barton L. L. Mandl M. Loy A. (Springer), 219–238. 10.1007/978-90-481-9204-5_10

[B17] Engel A. S. Engel S. A. (eds.). (2009). A field guide for the karst of Carter Caves State Resort Park and the surrounding area, northeastern Kentucky, in Select Field Guides to Cave and Karst Lands of the United States, Vol. 15 (Leesburg, VA: Karst Waters Institute Special Publication), 87–106.

[B18] Engel A. S. Northup D. E. (2008). Caves and karst as model systems for advancing the microbial sciences, in Frontiers of Karst Research, eds Martin J. B. White W. W. (Leesburg: Karst Waters Institute Special Publication), 37–48.

[B19] Engel A. S. Randall K. W. (2011). Experimental evidence for microbially mediated carbonate dissolution from the saline water zone of the Edwards Aquifer, Central Texas. Geomicrobiol. J. 28, 313–327. 10.1080/01490451.2010.500197

[B20] Engel A. S. Stern L. A. Bennett P. C. (2004). Microbial contributions to cave formation: new insights into sulfuric acid speleogenesis. Geology 32, 369 10.1130/g20288.1

[B21] Farnleitner A. H. Wilhartitz I. Ryzinska G. Kirschner A. K. Stadler H. Burtscher M. M.. (2005). Bacterial dynamics in spring water of alpine karst aquifers indicates the presence of stable autochthonous microbial endokarst communities. Environ. Microbiol. 7, 1248–1259. 10.1111/j.1462-2920.2005.00810.x 16011762

[B22] Ferguson R. I. Church M. (2004). A simple universal equation for grain settling velocity. J. Sediment. Res. 74, 933–937.

[B23] Feris K. Ramsey P. Frazar C. Rillig M. Gannon J. Holben W. (2003). Structure and seasonal dynamics of hyporheic zone microbial communities in free-stone rivers of the estern United States. Microb. Ecol. 46, 200–215. 10.1007/BF03036883 14708745

[B24] Fierer N. Morse J. L. Berthrong S. T. Bernhardt E. S. Jackson R. B. (2007). Environmental controls on the landscape-scale biogeography of stream bacterial communities. Ecology 88, 2162–2173. 10.1890/06-1746.1 17918395

[B25] Fierer N. Nemergut D. Knight R. Craine J. M. (2010). Changes through time: integrating microorganisms into the study of succession. Res. Microbiol. 161, 635–642. 10.1016/j.resmic.2010.06.002 20599610

[B26] Flynn T. M. Sanford R. A. Bethke C. M. (2008). Attached and suspended microbial communities in a pristine confined aquifer. Water Resour. Res. 44, W07425–W07432. 10.1029/2007wr006633

[B27] Ford D. Williams P. D. (2013). Karst Hydrogeology and Geomorphology. Chichester: John Wiley & Sons.

[B28] Fox J. Weisberg S. (2011). An {R} Companion to Applied Regression. Thousand Oaks, CA: Sage.

[B29] Gallon R. K. Fournier J. (2013). G2Sd: Grain-size Statistics and Description of Sediment, 2.0 Edn.

[B30] Ghimire B. Deng Z. (2013). Hydrograph-based approach to modeling bacterial fate and transport in rivers. Water Res. 47, 1329–1343. 10.1016/j.watres.2012.11.051 23270670

[B31] Gibert J. Danielopol D. Stanford J. A. (1994). Groundwater Ecology. San Diego, CA: Academic Press.

[B32] Griebler C. Lueders T. (2009). Microbial biodiversity in groundwater ecosystems. Freshwat. Biol. 54, 649–677. 10.1111/j.1365-2427.2008.02013.x

[B33] Griebler C. Stein H. Kellermann C. Berkhoff S. Brielmann H. Schmidt S. (2010). Ecological assessment of groundwater ecosystems – Vision or illusion? Ecol. Eng. 36, 1174–1190. 10.1016/j.ecoleng.2010.01.010

[B34] Hahn H. J. Fuchs A. (2009). Distribution patterns of groundwater communities across aquifer types in south-western Germany. Freshwat. Biol. 54, 848–860. 10.1111/j.1365-2427.2008.02132.x

[B35] Hazen T. C. Jimenes L. Lopez De Victoria G. Fliermans C. B. (1991). Comparison of bacteria from deep subsurface sediment and adjacent groundwater. Microb. Ecol. 22, 293–304. 24194344 10.1007/BF02540231

[B36] Huguet A. Vacher L. Relexans S. Saubusse S. Froidefond J. M. Parlanti E. (2009). Properties of fluorescent dissolved organic matter in the Gironde Estuary. Org. Geochem. 40, 706–719. 10.1016/j.orggeochem.2009.03.002

[B37] Hullar M. A. Kaplan L. A. Stahl D. A. (2006). Recurring seasonal dynamics of microbial communities in stream habitats. Appl. Environ. Microbiol. 72, 713–722. 10.1128/AEM.72.1.713-722.2006 16391111 PMC1352240

[B38] Kindt R. (2014). BiodiversityR: GUI for Biodiversity, Suitability and Community Ecology Analysis.

[B39] Kunin V. Engelbrektson A. Ochman H. Hugenholtz P. (2010). Wrinkles in the rare biosphere: pyrosequencing errors can lead to artificial inflation of diversity estimates. Environ. Microbiol. 12, 118–123. 10.1111/j.1462-2920.2009.02051.x 19725865

[B40] Lakowicz J. R. (2007). Principles of Fluorescence Spectroscopy. New York, NY: Springer Science & Business Media.

[B41] Lear G. Anderson M. J. Smith J. P. Boxen K. Lewis G. D. (2008). Spatial and temporal heterogeneity of the bacterial communities in stream epilithic biofilms. FEMS Microbiol. Ecol. 65, 463–473. 10.1111/j.1574-6941.2008.00548.x 18637965

[B42] Lehman R. M. (2007). Understanding of aquifer microbiology is tightly linked to sampling approaches. Geomicrobiol. J. 24, 331–341. 10.1080/01490450701456941

[B43] Lian B. Yuan D. Liu Z. (2011). Effect of microbes on karstification in karst ecosystems. Chin. Sci. Bull. 56, 3743–3747. 10.1007/s11434-011-4648-z

[B44] Lyautey E. Jackson C. R. Cayrou J. Rols J. L. Garabetian F. (2005). Bacterial community succession in natural river biofilm assemblages. Microb. Ecol. 50, 589–601. 10.1007/s00248-005-5032-9 16341639

[B45] McKnight D. M. Boyer E. W. Westerhoff P. K. Doran P. T. Kulbe T. Anderson D. T. (2001). Spectrofluorometric characterization of dissolved organic matter for indication of precursor organic material and aromaticity. Limnol. Oceanogr. 46, 38–48. 10.4319/lo.2001.46.1.0038

[B46] McMurdie P. J. Holmes S. (2013). phyloseq: an R package for reproducible interactive analysis and graphics of microbiome census data. PLoS ONE 8:e61217. 10.1371/journal.pone.0061217 23630581 PMC3632530

[B47] Morasch B. (2013). Occurrence and dynamics of micropollutants in a karst aquifer. Environ. Pollut. 173, 133–137. 10.1016/j.envpol.2012.10.014 23202643

[B48] Nico G. Daniel H. Rossi P. (2006). Review: Microbial biocenoses in pristine aquifers and an assessment of investigative methods. Hydrogeol. J. 14, 926–941. 10.1007/s10040-005-0009-9

[B49] Ohno T. (2002). Fluorescence inner-filtering correction for determining the humification index of dissolved organic matter. Envion. Sci. Technol. 38, 742–746. 10.1021/es0155276 11878392

[B50] Oksanen J. Blanchet F. Kindt R. Legendre P. O'hara R. Simpson G. (2013). Vegan: Community Ecology Package.

[B51] Ortiz M. Legatzki A. Neilson J. W. Fryslie B. Nelson W. M. Wing R. A.. (2014). Making a living while starving in the dark: metagenomic insights into the energy dynamics of a carbonate cave. ISME J. 8, 478–491. 10.1038/ismej.2013.159 24030597 PMC3906820

[B52] Pronk M. Goldscheider N. Zopfi J. (2008). Microbial communities in karst groundwater and their potential use for biomonitoring. Hydrogeol. J. 17, 37–48. 10.1007/s10040-008-0350-x

[B53] Rehmann C. R. Soupir M. L. (2009). Importance of interactions between the water column and the sediment for microbial concentrations in streams. Water Res. 43, 4579–4589. 10.1016/j.watres.2009.06.049 19615712

[B54] Riemann L. Leitet C. Pommier T. Simu K. Holmfeldt K. Larsson U.. (2008). The native bacterioplankton community in the central baltic sea is influenced by freshwater bacterial species. Appl. Environ. Microbiol. 74, 503–515. 10.1128/AEM.01983-07 18039821 PMC2223248

[B55] Shabarova T. Pernthaler J. (2010). Karst pools in subsurface environments: collectors of microbial diversity or temporary residence between habitat types. Environ. Microbiol. 12, 1061–1074. 10.1111/j.1462-2920.2009.02151.x 20132276

[B56] Shabarova T. Villiger J. Morenkov O. Niggemann J. Dittmar T. Pernthaler J. (2014). Bacterial community structure and dissolved organic matter in repeatedly flooded subsurface karst water pools. FEMS Microbiol. Ecol. 89, 111–126. 10.1111/1574-6941.12339 24716603

[B57] Shabarova T. Widmer F. Pernthaler J. (2013). Mass effects meet species sorting: transformations of microbial assemblages in epiphreatic subsurface karst water pools. Environ. Microbiol. 15, 2476–2488. 10.1111/1462-2920.12124 23614967

[B58] Shade A. Caporaso J. G. Handelsman J. Knight R. Fierer N. (2013). A meta-analysis of changes in bacterial and archaeal communities with time. ISME J. 7, 1493–1506. 10.1038/ismej.2013.54 23575374 PMC3721121

[B59] Simon K. S. Pipan T. Culver D. C. (2007). A conceptual model of the flow and distribution of organic carbon in caves. J. Cave Karst Stud. 69, 279–284.

[B60] Simon K. S. Pipan T. Ohno T. Culver D. C. (2010). Spatial and temporal patterns in abundance and character of dissolved organic matter in two karst aquifers. Fundam. Appl. Limnol. 177, 81–92. 10.1127/1863-9135/2010/0177-0081

[B61] Thomas J. M. Ward C. H. (1992). Subsurface microbial ecology and bioremediation. J. Hazard. Mater. 32, 179–194.

[B62] Van Der Gucht K. Vandekerckhove T. Vloemans N. Cousin S. Muylaert K. Sabbe K.. (2005). Characterization of bacterial communities in four freshwater lakes differing in nutrient load and food web structure. FEMS Microbiol. Ecol. 53, 205–220. 10.1016/j.femsec.2004.12.006 16329941

[B63] Vesper D. J. Loop C. M. White W. B. (2001). Contaminant transport in karst aquifers. Theor. Appl. Karstol. 13, 101–111. 10.1007/s10040-003-0299-8

[B64] Vetrovsky T. Baldrian P. (2013). The variability of the 16S rRNA gene in bacterial genomes and its consequences for bacterial community analyses. PLoS ONE 8:e57923. 10.1371/journal.pone.0057923 23460914 PMC3583900

[B65] Wang Q. Garrity G. M. Tiedje J. M. Cole J. R. (2007). Naive Bayesian classifier for rapid assignment of rRNA sequences into the new bacterial taxonomy. Appl. Environ. Microbiol. 73, 5261–5267. 10.1128/AEM.00062-07 17586664 PMC1950982

[B66] Wey J. K. Jurgens K. Weitere M. (2012). Seasonal and successional influences on bacterial community composition exceed that of protozoan grazing in river biofilms. Appl. Environ. Microbiol. 78, 2013–2024. 10.1128/AEM.06517-11 22247162 PMC3298157

[B67] Wilhartitz I. C. Kirschner A. K. Stadler H. Herndl G. J. Dietzel M. Latal C.. (2009). Heterotrophic prokaryotic production in ultraoligotrophic alpine karst aquifers and ecological implications. FEMS Microbiol. Ecol. 68, 287–299. 10.1111/j.1574-6941.2009.00679.x 19490127 PMC3119429

[B68] Yagi J. M. Neuhauser E. F. Ripp J. A. Mauro D. M. Madsen E. L. (2010). Subsurface ecosystem resilience: long-term attenuation of subsurface contaminants supports a dynamic microbial community. ISME J. 4, 131–143. 10.1038/ismej.2009.101 19776766

[B69] Zhou Y. Kellermann C. Griebler C. (2012). Spatio-temporal patterns of microbial communities in a hydrologically dynamic pristine aquifer. FEMS Microbiol. Ecol. 81, 230–242. 10.1111/j.1574-6941.2012.01371.x 22452537

[B70] Zhu F. Massana R. Not F. Marie D. Vaulot D. (2005). Mapping of picoeucaryotes in marine ecosystems with quantitative PCR of the 18S rRNA gene. FEMS Microbiol. Ecol. 52, 79–92. 10.1016/j.femsec.2004.10.006 16329895

