# Supplementary material for: Bacterial diversity differences along an epigenic cave stream reveal evidence of community dynamics, succession, and stability
Source: Front Microbiol. 2015 Jul 21;6:729. doi: 10.3389/fmicb.2015.00729 (PMC4508600; doi:10.3389/fmicb.2015.00729)
Supplement: Supplementary file 1 [file DataSheet1.DOCX]

**Supplement Table 1**. **Summary of pyrosequencing data for each of the samples used in this study. Average seq. length after trimming and % Chimeric were calculated in five batches.**

| **Sample Name** | **Small Read Archive Run Accession Number** | **Number of Seqs. (raw)** | **Number of Seqs. After Trimming** | **Average Seq. Length After Trimming** | **% Chimeric** | **OTUs (95% sequence identity)** | **Shannon**  **Index** | **Chao1**  **Index** |
| --- | --- | --- | --- | --- | --- | --- | --- | --- |
| CCRB.13d1 | SAMN03451539 | 7969 | 7191 | 492.8 | 25.1 | 875 | 5.77 | 1023.23 |
| CCRB.13d2 | SAMN03451542 | 6438 | 5652 | 492.8 | 25.1 | 932 | 6.19 | 1113.24 |
| CCRB.13N1 | SAMN03451551 | 8940 | 8061 | 492.8 | 25.1 | 962 | 5.84 | 1124.91 |
| CCRB.13N2 | SAMN03451554 | 7002 | 6276 | 492.8 | 25.1 | 867 | 5.78 | 1011.06 |
| CCRB.13O1A | SAMN03451561 | 15907 | 13349 | 471.1 | 38.1 | 1668 | 5.97 | 2130.62 |
| CCRB.13O1B | SAMN03451562 | 14262 | 11779 | 471.1 | 38.1 | 1392 | 5.14 | 1813.07 |
| CCRB.13O1C | SAMN03451563 | 17481 | 14329 | 471.1 | 38.1 | 1625 | 5.7 | 2063.34 |
| CCRB.13O2A | SAMN03451566 | 14414 | 12146 | 471.1 | 38.1 | 1772 | 6.21 | 2164.13 |
| CCRB.13O2B | SAMN03451567 | 13333 | 11369 | 471.1 | 38.1 | 1654 | 5.99 | 2046.55 |
| CCRB.13O2C | SAMN03451568 | 5310 | 4624 | 471.1 | 38.1 | 62 | 2.62 | 62.5 |
| CCRB.13S2A | SAMN03451573 | 12848 | 10622 | 471.1 | 38.1 | 1441 | 5.75 | 1913.68 |
| CCRB.13S2B | SAMN03451574 | 16369 | 13327 | 471.1 | 38.1 | 1523 | 5.74 | 1912.62 |
| CCRB.13S2C | SAMN03451575 | 9586 | 7755 | 471.1 | 38.1 | 1159 | 5.78 | 1520.17 |
| CCRB.13S3A | SAMN03451577 | 13941 | 11884 | 471.1 | 38.1 | 1758 | 6.13 | 2147.56 |
| CCRB.13S3B | SAMN03451578 | 16328 | 13889 | 471.1 | 38.1 | 2322 | 6.78 | 2661.2 |
| CCRB.13S3C | SAMN03451579 | 14418 | 12319 | 471.1 | 38.1 | 1767 | 5.94 | 2228.02 |
| CCRS.13A1 | SAMN03451533 | 4104 | 3731 | 469.9 | 34.6 | 258 | 3.44 | 294.85 |
| CCRS.13A2 | SAMN03451535 | 13143 | 11881 | 469.9 | 34.6 | 207 | 4.6 | 212.27 |
| CCRS.13A3 | SAMN03451537 | 3460 | 3099 | 469.9 | 34.6 | 230 | 6.72 | 248 |
| CCRS.13d1 | SAMN03451540 | 6022 | 5350 | 469.9 | 34.6 | 1214 | 4.27 | 1331.75 |
| CCRS.13d2 | SAMN03451543 | 3663 | 3176 | 469.9 | 34.6 | 131 | 4.71 | 142.4 |
| CCRS.13N1 | SAMN03451552 | 1736 | 1497 | 469.9 | 34.6 | 203 | 6.76 | 225.67 |
| CCRS.13N2 | SAMN03451555 | 6021 | 1693 | 469.9 | 34.6 | 1226 | 4.85 | 1389.2 |
| CCRS.13N3 | SAMN03451557 | 1905 | 5182 | 469.9 | 34.6 | 246 | 4.51 | 270.05 |
| CCRS.13O1 | SAMN03451559 | 2581 | 2182 | 469.9 | 34.6 | 186 | 5.09 | 205.33 |
| CCRS.13O2 | SAMN03451564 | 4805 | 4243 | 469.9 | 34.6 | 289 | 4.35 | 306 |
| CCRS.13O3 | SAMN03451569 | 1561 | 1359 | 469.9 | 34.6 | 166 | 4.65 | 190.8 |
| CCRS.13S1 | SAMN03451571 | 2520 | 2222 | 469.9 | 34.6 | 213 | 6.69 | 273.05 |
| CCRS.13S2 | SAMN03451572 | 14811 | 13077 | 469.9 | 34.6 | 1633 | 5.78 | 1698.35 |
| CCRW.13A1 | SAMN03451534 | 5120 | 4521 | 492.8 | 25.1 | 918 | 6.3 | 1063.75 |
| CCRW.13A2 | SAMN03451536 | 5718 | 5093 | 492.8 | 25.1 | 1030 | 6.42 | 1179.08 |
| CCRW.13A3 | SAMN03451538 | 4896 | 4261 | 492.8 | 25.1 | 836 | 6.08 | 935.23 |
| CCRW.13D1 | SAMN03451541 | 15327 | 13513 | 495.6 | 29.2 | 2117 | 6.33 | 2748.12 |
| CCRW.13D2 | SAMN03451544 | 17377 | 15459 | 495.6 | 29.2 | 2399 | 6.44 | 3139.65 |
| CCRW.13D3 | SAMN03451545 | 19492 | 17461 | 495.6 | 29.2 | 2684 | 6.7 | 3117.5 |
| CCRW.13JA1 | SAMN03451546 | 14537 | 12877 | 492.9 | 25.1 | 2139 | 6.61 | 2393.11 |
| CCRW.13JA3 | SAMN03451547 | 14016 | 12026 | 492.9 | 25.1 | 1787 | 6.37 | 1999.21 |
| CCRW.13JB1 | SAMN03451548 | 16038 | 14340 | 492.9 | 25.1 | 2388 | 6.78 | 2623.98 |
| CCRW.13JB3 | SAMN03451549 | 15484 | 13593 | 492.9 | 25.1 | 2032 | 6.48 | 2303.13 |
| CCRW.13JC1 | SAMN03451550 | 22010 | 19737 | 492.9 | 25.1 | 1876 | 5.21 | 2106.68 |
| CCRW.13N1 | SAMN03451553 | 22412 | 19960 | 495.6 | 29.2 | 2184 | 5.03 | 2672.43 |
| CCRW.13N2 | SAMN03451556 | 6823 | 6012 | 495.6 | 29.2 | 1676 | 6.66 | 2410.48 |
| CCRW.13N3 | SAMN03451558 | 17428 | 15493 | 495.6 | 29.2 | 1746 | 5.18 | 2272.56 |
| CCRW.13O1 | SAMN03451560 | 6546 | 5881 | 492.8 | 25.1 | 785 | 5.5 | 924.38 |
| CCRW.13O2 | SAMN03451565 | 8481 | 7597 | 492.8 | 25.1 | 1188 | 5.91 | 1299.04 |
| CCRW.13O3 | SAMN03451570 | 8331 | 7014 | 492.8 | 25.1 | 851 | 5.38 | 979.13 |
| CCRW.13S3 | SAMN03451576 | 8333 | 7513 | 492.8 | 25.1 | 781 | 4.81 | 905.05 |
| CCRW.13SF3 | SAMN03451580 | 10862 | 9876 | 492.8 | 25.1 | 513 | 3.53 | 606.31 |
| CCRW.13SL3 | SAMN03451581 | 26016 | 23437 | 495.6 | 29.2 | 1924 | 4.98 | 2204.79 |
| CCRB.13d1 | SAMN03451539 | 7969 | 7191 | 492.8 | 25.1 | 875 | 5.77 | 1023.23 |

**Supplement Table 2. . Results from ANOVA of CDOM fluorescence index by month and by location.**

|  |  | HIX | BIX | FI |
| --- | --- | --- | --- | --- |
| Month | F ratio | 76.93 | 0.81 | 12.78 |
|  | p-value | 3.43E-09 | 0.57 | 1.23E-04 |
| Location | F ratio | 0.47 | 1.18 | 0.86 |
|  | p-value | 0.63 | 0.33 | 0.44 |

Supplemental Figure 1. (Top) Bio-Traps® deployed at the upstream location in Cascade Cave System. (Bottom) Bio-Traps® deployed at the downstream location in Cascade Cave System.

**
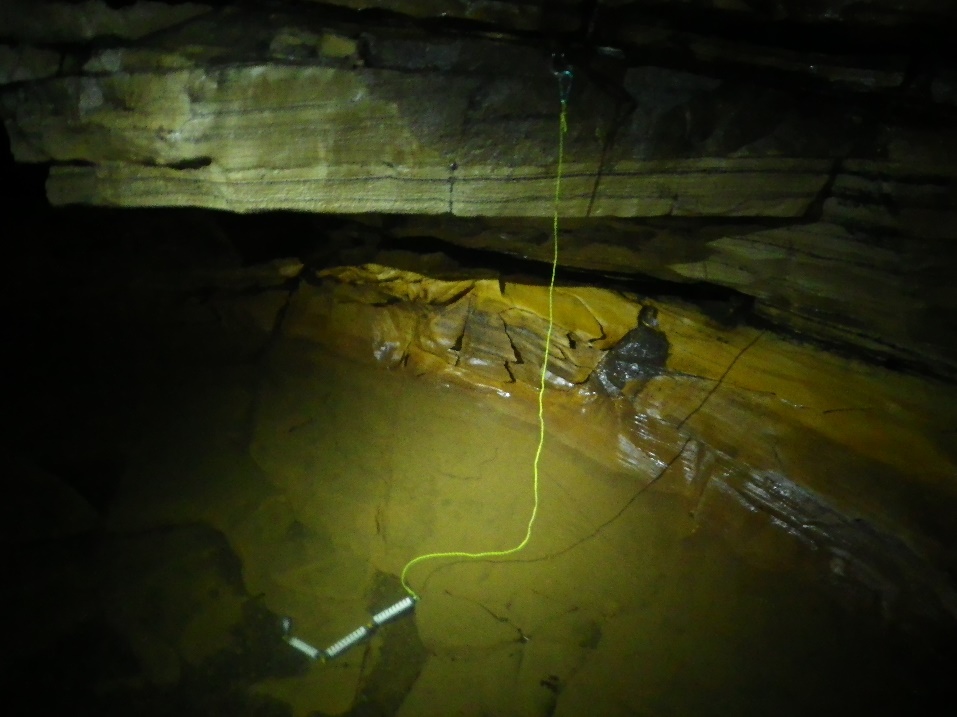

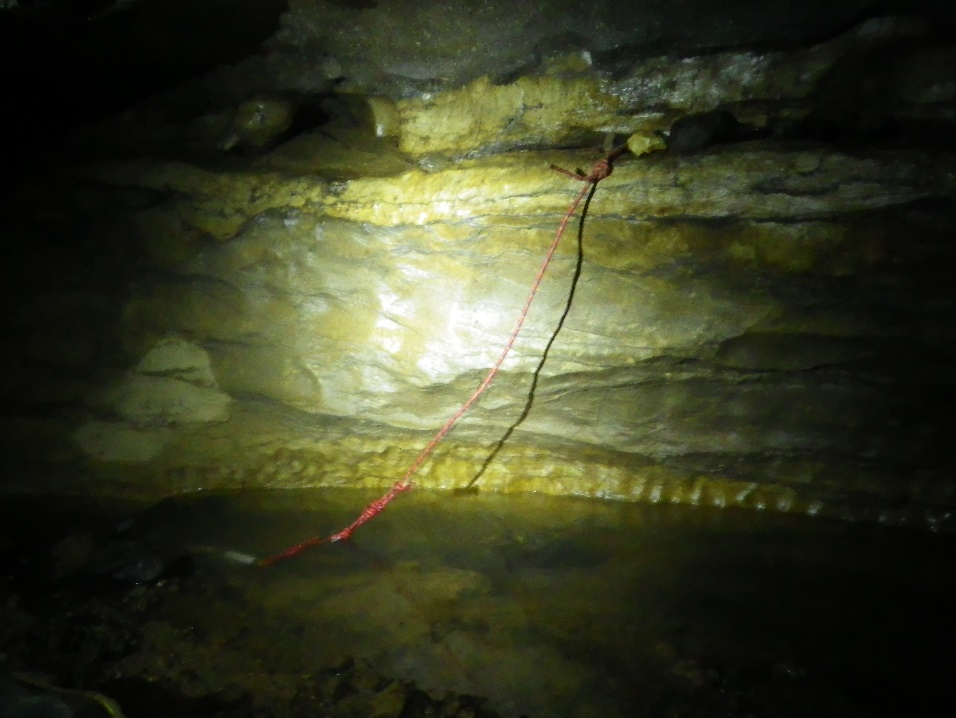
Supplemental Figure 2. Precipitation events over a 5 month period from the Olive Hill citizen scientist. Each vertical line represents the date of a sample event. Stream discharge measured at the downstream location of the Cascade Cave system. All non-precipitation events have been removed. Stream discharge rates below detection limit are not plotted.**

**
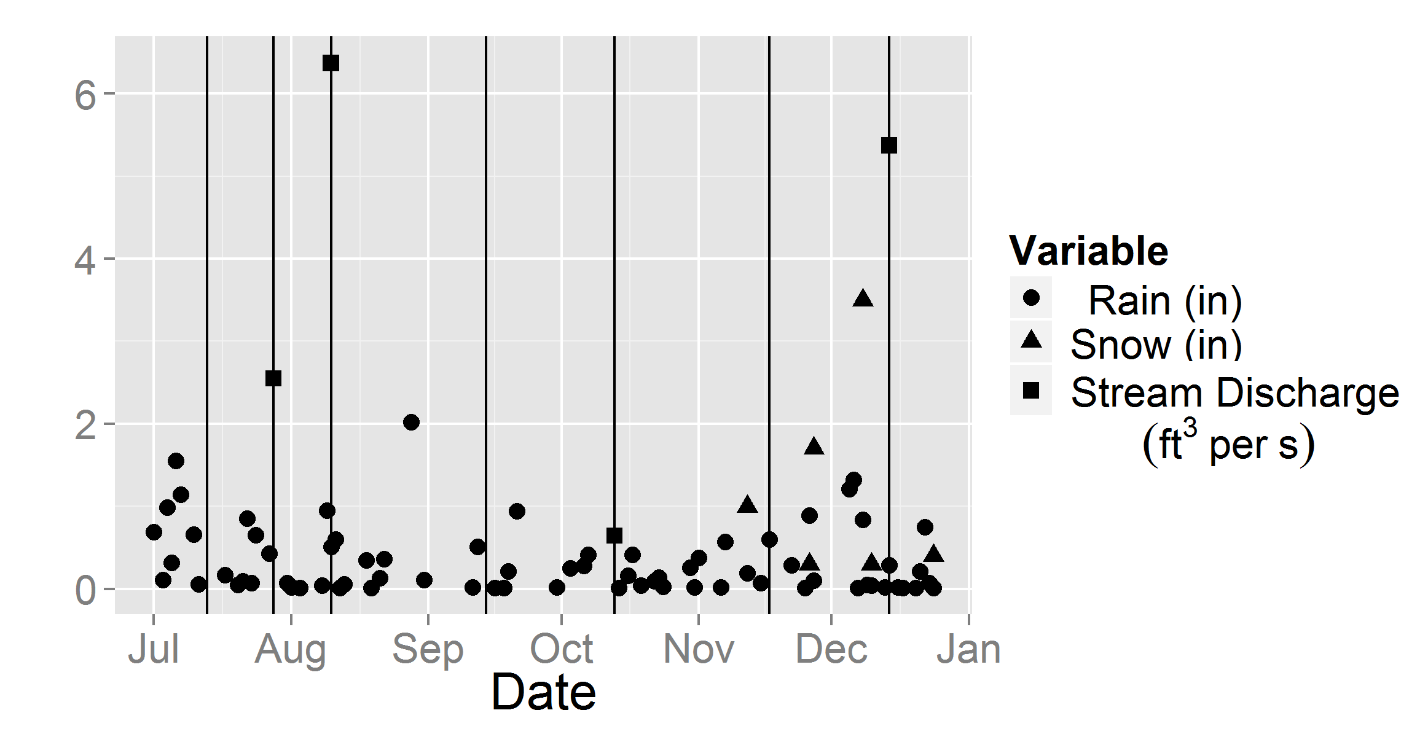
**

**Supplemental Figure 3. Rarefaction curves generated by QIIME using the Chao1 diversity metric. The calculation is cut after 7000 sequences. The samples with the lowest diversity are the sediment samples.
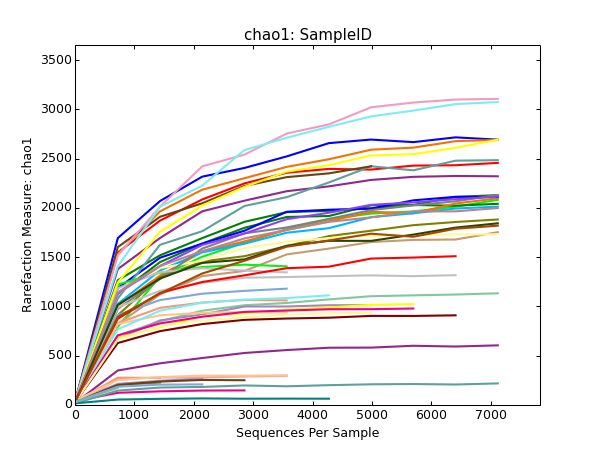
**

**Supplemental Figure 4. Percent grain size distribution of all sediment samples. Produced with the G2SD package for R.
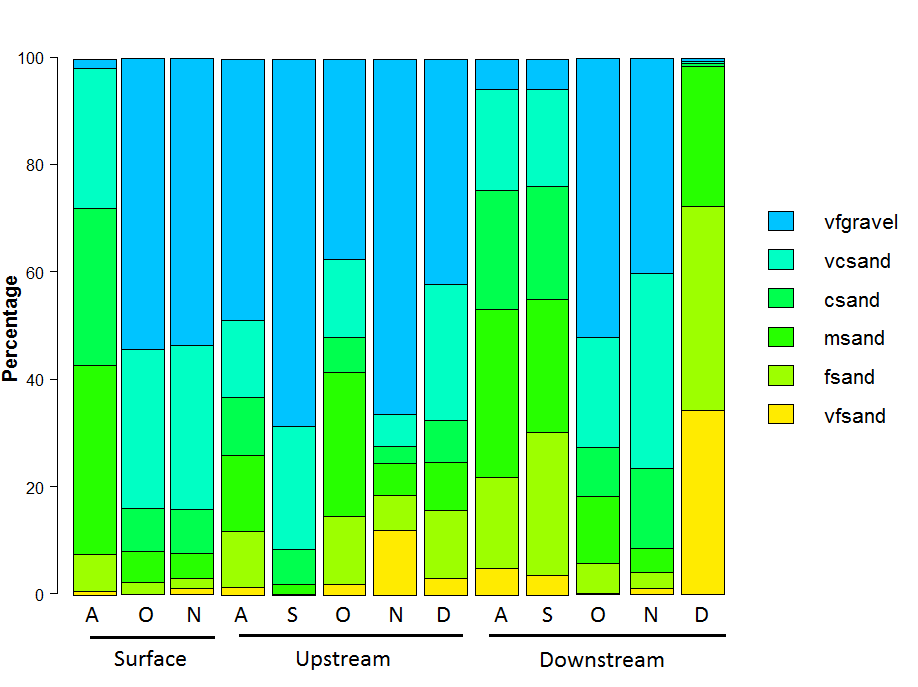
**

# R Scripts

This is an R Markdown document.

require(grid) #this is only required for the envfit arrows.

## Loading required package: grid

require(plyr) #make sure plyr is loaded b4 dplyr

require(dplyr) #data formatting and more

require(phyloseq) #OTU analysis

require(reshape2) #to get data into long format

require(ggplot2) #plotting

require(RColorBrewer) #ColorBrewer palettes

require(vegan) #Community Ecology Package: Ordination, Diversity and Dissimilarities

require(rmarkdown) #to make this script document

require(knitr) #to make this script document

require(BiodiversityR)

# Load in data to a phyloseq object

#import qiime biom with taxonomy and tre file
taxvec1 = c("k__Bacteria", "p__Firmicutes", "c__Bacilli", "o__Bacillales", "f__Staphylococcaceae")
mydata<-import_biom("otu_table_mc2_w_tax.biom","rep_set.tre",parseFunction=parse_taxonomy_greengenes)

## Warning in parseFunction(i$metadata$taxonomy): No greengenes prefixes were found.
## Consider using parse_taxonomy_default() instead if true for all OTUs.
## Dummy ranks may be included among taxonomic ranks now.

parse_taxonomy_greengenes(taxvec1)
#import mapping file
qiimedata <- import_qiime_sample_data("metadata_mapping.txt")
#merge them together
data <- merge_phyloseq(mydata, qiimedata)

Run numbers for general stats about our phyloseq object and full data set

#How many unique Genera are there?
taxa<-as.data.frame(tax_table(data))
#How many classified OTUs do we have?
apply(taxa, 2, function(x) length(which(!is.na(x))))
#How many unclassified OTUs do we have?
apply(taxa, 2, function(x) length(which(is.na(x))))

#How many sequences do we have?
sum(otu_table(data))

#How many OTUs do we have in each sample?
otus<-as.data.frame(otu_table(data))
head(otus)
num_otus<-as.data.frame(colSums(otus != 0))

#Let's see which are the most abundant Classes
class.sum <- tapply(taxa_sums(data), tax_table(data)[, "Class"], sum, na.rm = TRUE)
class.sum.table<-as.data.frame(class.sum)
class.sum.table$frac.abund<-(class.sum.table$class.sum/sum(class.sum.table$class.sum))*100
class.sum.table<-sort(class.sum.table$frac.abund, TRUE)
class.sum.table<-as.data.frame(class.sum.table)

OTU similarity table analyses

###############water
water <- subset_samples(data, Type == "Water")

#####fort falls
ff_w <- subset_samples(water, Location == "Surface")
shared_w_ff2<-filter_taxa(ff_w, function(x) (sum(x) !=0 ), TRUE)
shared_w_ff<-filter_taxa(ff_w, function(x) all(x !=0 ), TRUE)

##OTU and tax shared tables
shared_w_ff_1<-cbind(as.data.frame(otu_table(shared_w_ff)),as.data.frame(tax_table(shared_w_ff)))
shared_w_ff_1$otu<-rownames(shared_w_ff_1)
head(shared_w_ff_1)
shared_w_ff_1_melt<-melt(shared_w_ff_1)

## Using Kingdom, Phylum, Class, Order, Family, Genus, Species, Rank1, otu as id variables

head(shared_w_ff_1_melt)

#total number of seqs
num_seqs_w_ff<-sum(as.data.frame(otu_table(ff_w)))

#Add our other vaiables, but really we just need month
melt_vars1<-as.data.frame(cbind("sample"=as.character(qiimedata$Sample.ID),
 "type"=as.character(qiimedata$Type),
 "location"=as.character(qiimedata$Location),
 "month"=as.character(qiimedata$Month)))

rownames(melt_vars1)<-melt_vars1$sample
head(melt_vars1)

#use merge to add metadata to melted similarity data frame
shared_w_ff_1_melt2<-merge(shared_w_ff_1_melt,melt_vars1, by.x = 10, by.y = 0, all.x= TRUE)
str(shared_w_ff_1_melt2)

w_ff_melt<-shared_w_ff_1_melt2[,c(1,3:4,10:11,13:14)]
head(w_ff_melt)

w_ff_melt2<-w_ff_melt %>%
 group_by(Phylum,Class,type,location) %>%
 summarise(abundance=sum(value)/num_seqs_w_ff*100,
 OTUs=n()/7)

#####Upstream
j_w <- subset_samples(water, Location == "Upstream")
shared_w_j<-filter_taxa(j_w, function(x) all(x !=0 ), TRUE)
shared_w_j2<-filter_taxa(j_w, function(x) (sum(x) !=0 ), TRUE)

##OTU and tax shared tables
shared_w_j_1<-cbind(as.data.frame(otu_table(shared_w_j)),as.data.frame(tax_table(shared_w_j)))
shared_w_j_1$otu<-rownames(shared_w_j_1)
head(shared_w_j_1)
shared_w_j_1_melt<-melt(shared_w_j_1)

## Using Kingdom, Phylum, Class, Order, Family, Genus, Species, Rank1, otu as id variables

head(shared_w_j_1_melt)

#total number of seqs
num_seqs_w_j<-sum(as.data.frame(otu_table(j_w)))

#use merge to add metadata to melted similarity data frame
shared_w_j_1_melt2<-merge(shared_w_j_1_melt,melt_vars1, by.x = 10, by.y = 0, all.x= TRUE)
str(shared_w_j_1_melt2)

w_j_melt<-shared_w_j_1_melt2[,c(1,3:4,10:11,13:14)]
head(w_j_melt)

w_j_melt2<-w_j_melt %>%
 group_by(Phylum,Class,type,location) %>%
 summarise(abundance=sum(value)/num_seqs_w_j*100,
 OTUs=n()/5)


#####lake room
lr_w <- subset_samples(water, Location == "Downstream")
shared_w_lr<-filter_taxa(lr_w, function(x) all(x !=0 ), TRUE)
shared_w_lr2<-filter_taxa(lr_w, function(x) (sum(x) !=0 ), TRUE)

##OTU and tax shared tables
shared_w_lr_1<-cbind(as.data.frame(otu_table(shared_w_lr)),as.data.frame(tax_table(shared_w_lr)))
shared_w_lr_1$otu<-rownames(shared_w_lr_1)
head(shared_w_lr_1)
shared_w_lr_1_melt<-melt(shared_w_lr_1)

## Using Kingdom, Phylum, Class, Order, Family, Genus, Species, Rank1, otu as id variables

head(shared_w_lr_1_melt)

#total number of seqs
num_seqs_w_lr<-sum(as.data.frame(otu_table(lr_w)))


#use merge to add metadata to melted similarity data frame
shared_w_lr_1_melt2<-merge(shared_w_lr_1_melt,melt_vars1, by.x = 10, by.y = 0, all.x= TRUE)
str(shared_w_lr_1_melt2)

w_lr_melt<-shared_w_lr_1_melt2[,c(1,3:4,10:11,13:14)]
head(w_lr_melt)

w_lr_melt2<-w_lr_melt %>%
 group_by(Phylum,Class,type,location) %>%
 summarise(abundance=sum(value)/num_seqs_w_lr*100,
 OTUs=n()/8)


###############biotrap
biotrap <- subset_samples(data, Type== "Bio-Trap¨")

#####Upstream
j_bio <- subset_samples(biotrap, Location == "Upstream")
shared_b_j<-filter_taxa(j_bio, function(x) all(x !=0 ), TRUE)
shared_b_j2<-filter_taxa(j_bio, function(x) (sum(x) !=0 ), TRUE)

##OTU and tax shared tables
shared_b_j_1<-cbind(as.data.frame(otu_table(shared_b_j)),as.data.frame(tax_table(shared_b_j)))
shared_b_j_1$otu<-rownames(shared_b_j_1)
head(shared_b_j_1)
shared_b_j_1_melt<-melt(shared_b_j_1)

## Using Kingdom, Phylum, Class, Order, Family, Genus, Species, Rank1, otu as id variables

head(shared_b_j_1_melt)

#total number of seqs
num_seqs_b_j<-sum(as.data.frame(otu_table(j_bio)))

#use merge to add metadata to melted similarity data frame
shared_b_j_1_melt2<-merge(shared_b_j_1_melt,melt_vars1, by.x = 10, by.y = 0, all.x= TRUE)
str(shared_b_j_1_melt2)

b_j_melt<-shared_b_j_1_melt2[,c(1,3:4,10:11,13:14)]
head(b_j_melt)

b_j_melt2<-b_j_melt %>%
 group_by(Phylum,Class,type,location) %>%
 summarise(abundance=sum(value)/num_seqs_b_j*100,
 OTUs=n()/7)


#####lake room
lr_bio <- subset_samples(biotrap, Location == "Downstream")
shared_b_lr<-filter_taxa(lr_bio, function(x) all(x !=0 ), TRUE)
shared_b_lr2<-filter_taxa(lr_bio, function(x) (sum(x) !=0 ), TRUE)

##OTU and tax shared tables
shared_b_lr_1<-cbind(as.data.frame(otu_table(shared_b_lr)),as.data.frame(tax_table(shared_b_lr)))
shared_b_lr_1$otu<-rownames(shared_b_lr_1)
head(shared_b_lr_1)
shared_b_lr_1_melt<-melt(shared_b_lr_1)

## Using Kingdom, Phylum, Class, Order, Family, Genus, Species, Rank1, otu as id variables

head(shared_b_lr_1_melt)

#total number of seqs
num_seqs_b_lr<-sum(as.data.frame(otu_table(lr_bio)))

#use merge to add metadata to melted similarity data frame
shared_b_lr_1_melt2<-merge(shared_b_lr_1_melt,melt_vars1, by.x = 10, by.y = 0, all.x= TRUE)
str(shared_b_lr_1_melt2)

b_lr_melt<-shared_b_lr_1_melt2[,c(1,3:4,10:11,13:14)]
head(b_lr_melt)

 b_lr_melt2<-b_lr_melt %>%
 group_by(Phylum,Class,type,location) %>%
 summarise(abundance=sum(value)/num_seqs_b_lr*100,
 OTUs=n()/8)


##############sed
sediment <- subset_samples(data, Type == "Sediment")

#####fort falls
ff_s <- subset_samples(sediment, Location == "Surface")
shared_s_ff<-filter_taxa(ff_s, function(x) all(x !=0 ), TRUE)
shared_s_ff2<-filter_taxa(ff_s, function(x) (sum(x) !=0 ), TRUE)

##OTU and tax shared tables
shared_s_ff_1<-cbind(as.data.frame(otu_table(shared_s_ff)),as.data.frame(tax_table(shared_s_ff)))
shared_s_ff_1$otu<-rownames(shared_s_ff_1)
head(shared_s_ff_1)
shared_s_ff_1_melt<-melt(shared_s_ff_1)

## Using Kingdom, Phylum, Class, Order, Family, Genus, Species, Rank1, otu as id variables

head(shared_s_ff_1_melt)

#total number of seqs
num_seqs_s_ff<-sum(as.data.frame(otu_table(ff_s)))


#use merge to add metadata to melted similarity data frame
shared_s_ff_1_melt2<-merge(shared_s_ff_1_melt,melt_vars1, by.x = 10, by.y = 0, all.x= TRUE)
str(shared_s_ff_1_melt2)

s_ff_melt<-shared_s_ff_1_melt2[,c(1,3:4,10:11,13:14)]
head(s_ff_melt)

s_ff_melt2<-s_ff_melt %>%
 group_by(Phylum,Class,type,location) %>%
 summarise(abundance=sum(value)/num_seqs_s_ff*100,
 OTUs=n()/3)


#####Upstream
j_s <- subset_samples(sediment, Location == "Upstream")
shared_s_j<-filter_taxa(j_s, function(x) all(x !=0 ), TRUE)
shared_s_j2<-filter_taxa(j_s, function(x) (sum(x) !=0 ), TRUE)

##OTU and tax shared tables
shared_s_j_1<-cbind(as.data.frame(otu_table(shared_s_j)),as.data.frame(tax_table(shared_s_j)))
shared_s_j_1$otu<-rownames(shared_s_j_1)
head(shared_s_j_1)
shared_s_j_1_melt<-melt(shared_s_j_1)

## Using Kingdom, Phylum, Class, Order, Family, Genus, Species, Rank1, otu as id variables

head(shared_s_j_1_melt)

#total number of seqs
num_seqs_s_j<-sum(as.data.frame(otu_table(j_s)))


#use merge to add metadata to melted similarity data frame
shared_s_j_1_melt2<-merge(shared_s_j_1_melt,melt_vars1, by.x = 10, by.y = 0, all.x= TRUE)
str(shared_s_j_1_melt2)

s_j_melt<-shared_s_j_1_melt2[,c(1,3:4,10:11,13:14)]
head(s_j_melt)

s_j_melt2<-s_j_melt %>%
 group_by(Phylum,Class,type,location) %>%
 summarise(abundance=sum(value)/num_seqs_s_j*100,
 OTUs=n()/5)

#####lake room
lr_s <- subset_samples(sediment, Location == "Downstream")
shared_s_lr<-filter_taxa(lr_s, function(x) all(x !=0 ), TRUE)
shared_s_lr2<-filter_taxa(lr_s, function(x) (sum(x) !=0 ), TRUE)

##OTU and tax shared tables
shared_s_lr_1<-cbind(as.data.frame(otu_table(shared_s_lr)),as.data.frame(tax_table(shared_s_lr)))
shared_s_lr_1$otu<-rownames(shared_s_lr_1)
head(shared_s_lr_1)
shared_s_lr_1_melt<-melt(shared_s_lr_1)

## Using Kingdom, Phylum, Class, Order, Family, Genus, Species, Rank1, otu as id variables

head(shared_s_lr_1_melt)

#total number of seqs
num_seqs_s_lr<-sum(as.data.frame(otu_table(lr_s)))


#use merge to add metadata to melted similarity data frame
shared_s_lr_1_melt2<-merge(shared_s_lr_1_melt,melt_vars1, by.x = 10, by.y = 0, all.x= TRUE)
str(shared_s_lr_1_melt2)

s_lr_melt<-shared_s_lr_1_melt2[,c(1,3:4,10:11,13:14)]
head(s_lr_melt)

s_lr_melt2<-s_lr_melt %>%
 group_by(Phylum,Class,type,location) %>%
 summarise(abundance=sum(value)/num_seqs_s_lr*100,
 OTUs=n()/5)


##################make our final table!
shared_table<-as.data.frame(rbind(s_ff_melt2,s_j_melt2,s_lr_melt2,b_j_melt2,b_lr_melt2,w_ff_melt2,w_j_melt2,w_lr_melt2))
shared_table_otus<-dcast(shared_table,Phylum+Class~type+location,value.var="OTUs")
shared_table_abund<-dcast(shared_table,Phylum+Class~type+location,value.var="abundance")

Anosim and Adonis analyses

#dissimilarity matrix
bray<-ordinate(data,distance="bray",method="NMDS")

#Adonis
bray_dist<-distance(data,"bray")

type<- adonis(bray_dist~sample_data(data)$Type,permutations=9999)
month<- adonis(bray_dist~sample_data(data)$Month,permutations=9999)
location<- adonis(bray_dist~sample_data(data)$Location,permutations=9999)
densityplot(permustats(location))

fi<- adonis(bray_dist~sample_data(data)$FI,permutations=9999)
hix<- adonis(bray_dist~sample_data(data)$HIX,permutations=9999)

# NMDS script

Data processing

data_filter= filter_taxa(data, function(x) sum(x > 3) > (0.2*length(x)), TRUE)
ntaxa(data)
ntaxa(data_filter)

#Standardize abundances to the median sequencing depth
total = median(sample_sums(data_filter))
standf = function(x, t=total) round(t * (x / sum(x)))
data_trans = transform_sample_counts(data_filter, standf)

#Filter the taxa using a cutoff of 2.0 for the Coefficient of Variation
data_trans_cv = filter_taxa(data_trans, function(x) sd(x)/mean(x) > 2.0, TRUE)
ntaxa(data_trans_cv)

# NMDS plot

taken from the excellent stackoverflow Q+A: <http://stackoverflow.com/questions/13794419/plotting-ordiellipse-function-from-vegan-package-onto-nmds-plot-created-in-ggplo>

#Ordinate NMDS using Bray, use stress plot to find the best dissimilarit/distance
nmds.bray <- ordinate(data,"NMDS","bray")
stressplot(nmds.bray)

as.data.frame(nmds.bray$points)

environ<-data.frame(sample_data(data)[,2:16])
str(environ)
##site data
sites <- data.frame(scores(nmds.bray, choices=c(1,2),display = c("sites"))) #dataframe of species scoes for plotting
head(sites)
sites$Location <- sample_data(data)$Location #otherwise factor doesn't drop unused levels and it will throw an error
sites$Type <- sample_data(data)$Type
sites$Month <- sample_data(data)$Month

#envfit
nmds.bray.envfit <- envfit(as.data.frame(nmds.bray$points),
 env = environ,na.rm=TRUE, perm = 999) #standard envfit
plot(nmds.bray)
plot(nmds.bray.envfit,p=0.05)

#ellipses
# function for ellipsess - just run this, is used later
veganCovEllipse <- function (cov, center = c(0, 0), scale = 1, npoints = 100)
{
 theta <- (0:npoints) * 2 * pi/npoints
 Circle <- cbind(cos(theta), sin(theta))
 t(center + scale * t(Circle %*% chol(cov)))
}

#data for ellipse, in this case using the management factor
df_ell.dune.management <- data.frame() #sets up a data frame before running the function.
for(g in levels(sites$Type)){
 df_ell.dune.management <- rbind(df_ell.dune.management, cbind(as.data.frame(with(sites [sites$Type==g,],
 veganCovEllipse(cov.wt(cbind(NMDS1,NMDS2),wt=rep(1/length(NMDS1),length(NMDS1)))$cov,center=c(mean(NMDS1),mean(NMDS2)))))
 ,Type=g))
}

# data for labelling the ellipse
NMDS.mean.biotrap=aggregate(sites[ ,c("NMDS1", "NMDS2")],
 list(group = sites$Type), mean)

# data for labelling the ellipse
NMDS.mean=aggregate(sites[,c("NMDS1", "NMDS2")],
 list(group = sites$Type), mean)


## finally plotting.
getPalette = colorRampPalette(brewer.pal(8, "Accent"))
#display.brewer.pal(8,"Dark2")

nmds2 <- ggplot(data = sites, aes(x = NMDS1, y = NMDS2))+ #sets up the plot. brackets around the entire thing to make it draw automatically
 geom_point(aes(x = NMDS1, y = NMDS2, shape=Type),size = 6) + #puts the site points in from the ordination, shape determined by site, size refers to size of point
 geom_path(data = df_ell.dune.management, aes(x = NMDS1, y = NMDS2, group = Type)) + #this is the ellipse, seperate ones by type. If you didn't change the "alpha" (the shade) then you need to keep the "group annotate("text",x = -0.70,y = 0.5,label="Filter",size=6) + #labels for the centroids - I haven't used this since we have a legend. but you could also dithc the legend, but plot will get v messy
 theme_bw(base_size = 15) +
 theme(axis.text.x=element_blank(),
 axis.text.y=element_blank(),axis.ticks=element_blank(),
 axis.title.x=element_blank(), axis.title.y=element_blank()
 )

ggsave(file="NMDS_plot.tiff",plot=nmds2,width=10,height=7,units="in",dpi=300)

# PCA/RDA plot

with help from <https://oliviarata.wordpress.com/2014/09/06/rda-in-ggplot2/>

require(grid) #this is only required for the envfit arrows.
require(plyr) #make sure plyr is loaded b4 dplyr
require(dplyr) #data formatting and more
require(phyloseq) #OTU analysis
require(reshape2) #to get data into long format
require(ggplot2) #plotting
require(RColorBrewer) #ColorBrewer palettes
require(vegan) #Community Ecology Package: Ordination, Diversity and Dissimilarities
require(rmarkdown) #to make this script document
require(knitr) #to make this script document
#create a veganified phyloseq object
veganotu <- function(physeq) {
 OTU <- otu_table(physeq)
 if (taxa_are_rows(OTU)) {
 OTU <- t(OTU)
 }
 OTU <- as(OTU, "matrix")
 return(OTU)
}


vegan_otu<-as.data.frame(veganotu(data_trans_cv))
sample.data<-data.frame(sample_data(data_trans_cv))

all.rda<- rda(vegan_otu~Type+HIX+FI, data=sample.data)
all.rda
PCAsignificance(all.rda,axes=4)
plot(all.rda)

scor = scores(all.rda, display=c("sp", "cn", "bp"), scaling=2)

# type centroids
type_numeric_centroids <- data.frame(scor$centroids)
type_numeric_centroids
type_numeric_centroids$type <- rownames(type_numeric_centroids)
type_numeric_centroids

#sites
site_scores <- data.frame(scores(all.rda)$sites)
site_scores$type <-sample_data(data_trans_cv)$Type
str(site_scores)

# arrows
type_continuous_arrows <- data.frame(scor$biplot)
type_continuous_arrows
type_continuous_arrows$type_class <- rownames(type_continuous_arrows) #turning rownames into a variable
type_continuous_arrows

mult <- attributes(scores(all.rda))$const # scaling for the arrows

RDA_plot <- ggplot(site_scores, aes(x = RDA1, y = RDA2))+
 theme_bw() +
 geom_point(aes(size = 3,shape=type)) +
 scale_shape_manual(values = c('Bio-Trap®' = 17, 'Water' = 16, 'Sediment'=15),guide="legend") +
 geom_segment(data = type_continuous_arrows,
 aes(x = 0, xend = mult * RDA1,
 y = 0, yend = mult * RDA2),
 arrow = arrow(length = unit(0.25, "cm")), colour = "grey") + #grid is required for arrow to work.
 geom_text(data = type_continuous_arrows,
 aes(x= (mult + mult/5) * RDA1, y = (mult + mult/5) * RDA2,
 label = type_class),
 size = 5,
 hjust = 0.5)


ggsave(plot = RDA_plot, file = "RDA.tiff",width=10,height=7,units="in",dpi=300)

## Warning: Removed 16 rows containing missing values (geom_point).

# Richness and Evenness plots

##reorder months
sample_data(data)$Month <- factor(sample_data(data)$Month, levels = c("July", "August","September","October", "November","December"),ordered=TRUE)
levels(sample_data(data)$Month)

richness_all<- plot_richness(data,measures =rbind("Observed","Chao1", "Shannon"),color="Month", shape="Location")
str(richness_all)
head(richness_all$data$variable)
richness<-as.data.frame(richness_all$data)

write.csv(richness,"richness_data.csv")

richness_table<-as.data.frame(cbind("Month"=as.character(richness_all$data$Month),"Location"=as.character(richness_all$data$Location),"Type"=as.character(richness_all$data$Type),"Index"=as.character(richness_all$data$variable),"value"=as.numeric(richness_all$data$value)))
str(richness_table)
richness_table$value<-as.numeric(as.character(richness_table$value))
richness_table$Month = with(richness_table, factor(Month, levels = c("July","August","September","October", "November","December"),ordered=TRUE))

Chao1 <- subset(richness_table, Index== "Chao1")
Shannon <- subset(richness_table, Index== "Shannon")
Observed<- subset(richness_table, Index== "Observed")

Chao1_plot<-ggplot(data=Chao1,aes(x=Month,y=value))+
 theme_grey(base_size = 20)+
 geom_point(size=6) +
 stat_smooth(method="loess",aes(group=1)) +
 facet_grid(Type~Location,scales="fixed") +
 theme(axis.text.x = element_text(angle = 45,vjust=1,hjust=1),axis.title.x=element_blank(),axis.title.y=element_blank()) +
 labs(title = "Chao1")
ggsave(file="chao1.tiff",plot=Chao1_plot,width=10,height=8,units="in",dpi=300)

Shannon_plot<-ggplot(data=Shannon,aes(x=Month,y=value))+
 theme_grey(base_size = 20)+
 geom_point(size=6) +
 stat_smooth(method="loess",aes(group=1)) +
 facet_grid(Type~Location,scales="fixed") +
 theme(axis.text.x = element_text(angle = 45,vjust=1,hjust=1),axis.title.x=element_blank(),axis.title.y=element_blank()) +
 labs(title = "Shannon")
ggsave(file="shannon.tiff",plot=Shannon_plot,width=10,height=8,units="in",dpi=300)

Observed_plot<-ggplot(data=Observed,aes(x=Month,y=value))+
 theme_grey(base_size = 20)+
 geom_point(size=6) +
 stat_smooth(method="loess",aes(group=1)) +
 facet_grid(Type~Location,scales="fixed") +
 theme(axis.text.x = element_text(angle = 45,vjust=1,hjust=1),axis.title.x=element_blank(),axis.title.y=element_blank()) +
 labs(title = "Observed")
ggsave(file="Observed_all.tiff",plot=Observed_plot,width=10,height=8,units="in",dpi=300)

# OTU tracking over time graphs

##################################water
water <- subset_samples(data, Type == "Water")
shared_w<-filter_taxa(water, function(x) all(x !=0 ), TRUE)
#16

#### water by location
#fort falls
ff_w <- subset_samples(water, Location == "Surface")
w_ff<-filter_taxa(ff_w, function(x) all(x !=0 ), TRUE)
shared_w_ff<-as.data.frame(tax_table(w_ff))
shared_w_ff_otus<-as.data.frame(otu_table(w_ff))
shared_w_ff$abundance<-rowSums(shared_w_ff_otus)
shared_w_ff$type<-"Water"
shared_w_ff$loc1<-"Surface"
shared_w_ff$loc2<-"Surface"
head(shared_w_ff)

#Upstream
j_w <- subset_samples(water, Location == "Upstream")
shared_w_j<-filter_taxa(j_w, function(x) all(x !=0 ), TRUE)

#How many sequences do we have?
sum(as.data.frame(otu_table(j_w)))
#create new normalized abundance. Normalized to the total number of sequences in each biotrap samples
j_w_trans<-transform_sample_counts(j_w, function(OTU) OTU/sum(OTU)*100)
shared_w_j<-filter_taxa(j_w_trans, function(x) all(x !=0 ), TRUE)

#for a plot, we need to melt this data to a long format
shared_w_j_otu_tax<-cbind(as.data.frame(otu_table(shared_w_j)),as.data.frame(tax_table(shared_w_j)))
shared_w_j_otu_tax$otu<-rownames(shared_w_j_otu_tax)
head(shared_w_j_otu_tax)
shared_wj_melt<-melt(shared_w_j_otu_tax)

## Using Kingdom, Phylum, Class, Order, Family, Genus, Species, Rank1, otu as id variables

head(shared_wj_melt)

#use merge to add metadata to melted similarity data frame
melt_shared_wj<-merge(shared_wj_melt,melt_vars1, by.x = 10, by.y = 0, all.x= TRUE)
head(melt_shared_wj)


#drop unsused levels
melt_shared_wj<-droplevels(melt_shared_wj)
#order months by time
months<-c("July","August","September","October","November","December")
melt_shared_wj$month<-factor(melt_shared_wj$month,levels=months)

#use only the most abundant OTUs
greater_than_four_wj<-subset(melt_shared_wj,subset=value>4,select=otu)
greater_than_four_wj_percent<-subset(melt_shared_wj, otu %in% greater_than_four_wj$otu)

#Average the replicates
#no replicates here
#create percent column as duplicate of value so we can combine datasets later
greater_than_four_wj_percent$percent<-greater_than_four_wj_percent$value

#lake room
lr_w <- subset_samples(water, Location == "Downstream")
w_lr<-filter_taxa(lr_w, function(x) all(x !=0 ), TRUE)

#How many sequences do we have?
sum(as.data.frame(otu_table(lr_w)))
#create new normalized abundance. Normalized to the total number of sequences in each biotrap samples
lr_w_trans<-transform_sample_counts(lr_w, function(OTU) OTU/sum(OTU)*100)
shared_w_lr<-filter_taxa(lr_w_trans, function(x) all(x !=0 ), TRUE)

#for a plot, we need to melt this data to a long format
shared_w_lr_otu_tax<-cbind(as.data.frame(otu_table(shared_w_lr)),as.data.frame(tax_table(shared_w_lr)))
shared_w_lr_otu_tax$otu<-rownames(shared_w_lr_otu_tax)
head(shared_w_lr_otu_tax)
shared_wlr_melt<-melt(shared_w_lr_otu_tax)

## Using Kingdom, Phylum, Class, Order, Family, Genus, Species, Rank1, otu as id variables

head(shared_wlr_melt)

#use merge to add metadata to melted similarity data frame
melt_shared_wlr<-merge(shared_wlr_melt,melt_vars1, by.x = 10, by.y = 0, all.x= TRUE)
head(melt_shared_wlr$month)


#drop unsused levels
#melt_shared_wlr<-droplevels(melt_shared_wlr)
#order months by time
months<-c("July","August","September","October","November","December")
melt_shared_wlr$month<-factor(melt_shared_wlr$month,levels=months)

#use only the most abundant OTUs
greater_than_four_wlr<-subset(melt_shared_wlr,subset=value>4,select=otu)
greater_than_four_wlr_percent<-subset(melt_shared_wlr, otu %in% greater_than_four_wlr$otu)

#Average the replicates
greater_than_four_wlr_percent2<-ddply(greater_than_four_wlr_percent, .(otu,month), transform, percent=mean(value),.drop=FALSE)


#Now let's put our locations together
water_greater_than_four<-rbind(greater_than_four_wlr_percent2,greater_than_four_wj_percent)

janky_comb<-ggplot(water_greater_than_four,aes(x=month,y=percent,group=otu,color=otu)) +
 geom_line(size=2) +
 facet_wrap(~location) +
 theme_bw(base_size=12) +
 theme(axis.title.x=element_blank(),panel.grid.major=element_line(colour = "darkgrey")) +
 ylab(("Sequence abundance (%) normalized by each sample")) +
 scale_color_manual(values=c("darkorchid3","dodgerblue4","darkolivegreen4","darkorange","cyan","firebrick2","deeppink4"))

ggsave("water_comb_order.tiff",janky_comb,width=10,height=5,units="in",dpi=300)

################################ biotrap by location
#Upstream
j_b <- subset_samples(biotrap, Location == "Upstream")
#How many sequences do we have?
sum(as.data.frame(otu_table(j_b)))
#create new normalized abundance. Normalized to the total number of sequences in each biotrap samples
j_b_trans<-transform_sample_counts(j_b, function(OTU) OTU/sum(OTU)*100)
shared_b_j<-filter_taxa(j_b_trans, function(x) all(x !=0 ), TRUE)

#for a plot, we need to melt this data to a long format
shared_b_j_otu_tax<-cbind(as.data.frame(otu_table(shared_b_j)),as.data.frame(tax_table(shared_b_j)))
shared_b_j_otu_tax$otu<-rownames(shared_b_j_otu_tax)
head(shared_b_j_otu_tax)
shared_bj_melt<-melt(shared_b_j_otu_tax)

## Using Kingdom, Phylum, Class, Order, Family, Genus, Species, Rank1, otu as id variables

head(shared_bj_melt)

#use merge to add metadata to melted similarity data frame
melt_shared_bj<-merge(shared_bj_melt,melt_vars1, by.x = 10, by.y = 0, all.x= TRUE)
head(melt_shared_bj)

#drop unsused levels
melt_shared_bj<-droplevels(melt_shared_bj)
#order months by time
months<-c("September","October","November","December")
melt_shared_bj$month<-factor(melt_shared_bj$month,levels=months)

#use only the most abundant OTUs
greater_than_four_otu<-subset(melt_shared_bj,subset=value>4,select=otu)
greater_than_four_percent<-subset(melt_shared_bj, otu %in% greater_than_four_otu$otu)

#Average the replicates
greater_than_four_percent2<-ddply(greater_than_four_percent, .(otu,month), transform, percent=mean(value),.drop=FALSE)

#lake room
lr_b <- subset_samples(biotrap, Location == "Downstream")
shared_b_lr<-filter_taxa(lr_b, function(x) all(x !=0 ), TRUE)
#How many sequences do we have?
sum(as.data.frame(otu_table(lr_b)))
#create new normalized abundance. Normalized to the total number of sequences in each biotrap samples
lr_b_trans<-transform_sample_counts(shared_b_lr, function(OTU) OTU/sum(OTU)*100)
#for a plot, we need to melt this data to a long format
shared_b_lr_otu_tax<-cbind(as.data.frame(otu_table(lr_b_trans)),as.data.frame(tax_table(lr_b_trans)))
shared_b_lr_otu_tax$otu<-rownames(shared_b_lr_otu_tax)
head(shared_b_lr_otu_tax)
shared_blr_melt<-melt(shared_b_lr_otu_tax)

## Using Kingdom, Phylum, Class, Order, Family, Genus, Species, Rank1, otu as id variables

head(shared_blr_melt)

#use merge to add metadata to melted similarity data frame
melt_shared_blr<-merge(shared_blr_melt,melt_vars1, by.x = 10, by.y = 0, all.x= TRUE)
head(melt_shared_blr)


#drop unsused levels
melt_shared_blr<-droplevels(melt_shared_blr)
#order months by time
months<-c("September","October","November","December")
melt_shared_blr$month<-factor(melt_shared_blr$month,levels=months)

#Pull out OTUs that are greater than 4% abundance
greater_than_four_otu_lr<-subset(melt_shared_blr,subset=value>4,select=otu)
greater_than_four_percent_lr<-subset(melt_shared_blr, otu %in% greater_than_four_otu_lr$otu)
greater_than_four_percent2_lr<-ddply(greater_than_four_percent_lr, .(otu,month), transform, percent=mean(value),.drop=FALSE)


#####Now let's combine them together
biotrap_shared<-rbind(greater_than_four_percent2_lr,greater_than_four_percent2)

jamky_bio<-ggplot(biotrap_shared,aes(x=month,y=percent,group=otu,color=otu)) +
 geom_line(size=2) +
 theme_bw(base_size=12) +
 facet_wrap(~location) +
 theme(axis.title.x=element_blank(),panel.grid.major=element_line(colour = "darkgrey")) +
 ylab(("Sequence abundance (%) normalized by each sample")) +
 scale_color_manual(values=c("khaki1","khaki4","lightgreen","thistle",
 "tomato","paleturquoise4"))
ggsave("biotrap_succession_class.tiff",jamky_bio,width=10,height=5,units="in",dpi=300)
